# Tools to measure barriers to medication management capacity in older adults: a scoping review

**DOI:** 10.1186/s12877-024-04893-7

**Published:** 2024-03-27

**Authors:** Bincy Baby, Annette McKinnon, Kirk Patterson, Hawa Patel, Rishabh Sharma, Caitlin Carter, Ryan Griffin, Catherine Burns, Feng Chang, Sara JT Guilcher, Linda Lee, Sara Abu Fadaleh, Tejal Patel

**Affiliations:** 1 School of Pharmacy, University of Waterloo, Waterloo, ON Canada; 2 Patient Advisor’s Network, Toronto, ON Canada; 3 National Research Council Canada, Ottawa, ON Canada; 4 Faculty of Engineering, University of Waterloo, Waterloo, ON Canada; 5 Leslie Dan Faculty of Pharmacy, University of Toronto, Toronto, ON Canada; 6 Department of Family Medicine, McMaster University, Hamilton, Canada

**Keywords:** Older adults, Medication management, Self-management, Adherence, Barriers

## Abstract

**Background:**

Medication management capacity is a crucial component of medication adherence, particularly among older adults. Various factors, including physical abilities, cognitive functions, sensory capabilities, motivational, and environmental factors, influence older adults' ability to manage medications. It is, therefore, crucial to identify appropriate tools that allow clinicians to determine which factors may impact medication management capacity and, consequently, nonadherence to medications.

**Purpose:**

1)To identify tools that measure physical, cognitive, sensory (vision, hearing, touch), motivational, and environmental barriers to medication self-management in older adults, and 2) to understand the extent to which these tools assess various barriers.

**Methods:**

The scoping review was conducted using Arksey and O'Malley's scoping review framework and the PRISMA Extension for Scoping Reviews checklist. In June 2022, the relevant literature was identified by searching PubMed (MEDLINE), Ovid Embase, Ovid IPA, EBSCOhost CINAHL, APA PsycINFO, and Scopus.

**Results and discussion:**

In total, 7235 studies were identified. Following the removal of duplicates, 4607 articles were screened by title and abstract, of which 4253 did not meet the inclusion criteria. Three reviewers reviewed the full texts of the remaining 354 articles; among them, 41 articles, 4 theses and 1 conference abstract met the inclusion criteria. From the included studies, 44 tools were identified that measured a combination of physical, cognitive, sensory, motivational, and environmental barriers (*n*=19) or only cognition (*n*=13), vision (*n*=5), environmental factors (*n*=3), auditory (*n*=1), and motivational factors (*n*=1). The review also examined the psychometric properties of the identified tools and found that most of them had reported validity and reliability data. Several tools have demonstrated promise in assessing a combination of barriers with validity and reliability. These tools include the Self-Medication Assessment Tool (SMAT), ManageMed Screening (MMS), Self-Medication Risk Assessment Tool (RAT), HOME-Rx revised, and Medication Management Ability Assessment (MMAA).

**Conclusion:**

This scoping review identified 44 validated tools to measure various challenges that older adults encounter with medication management. However, no tool measures all five barriers (physical, cognitive, sensory, motivational, and environmental) to medication-taking at home. Therefore, utilizing a combination of tools would be most appropriate to measure these different aspects comprehensively. Further research is needed to develop a new comprehensive tool that simultaneously measures various barriers to medication self-management.

**Supplementary Information:**

The online version contains supplementary material available at 10.1186/s12877-024-04893-7.

## Introduction

In individuals aged 60 years and above, there is a higher prevalence of multiple chronic conditions, including diabetes, hypertension, heart disease, stroke, and cancer, compared to younger age groups [1]. According to a report by the National Council on Aging, “approximately 92% of older adults have at least one chronic disease, and 77% have at least two chronic diseases” [2]. These chronic diseases, along with associated disabilities, can result in complex medication regimens and an increased risk of functional impairment, presenting significant challenges in medication management capacity [3]. Additionally, the burden of handling medications, especially within the context of multi-morbidity and complex medications regimens, introduces an added layer of complexity to the day-to-day lives of older people, and these burdens can also influence their capacity to manage medications [4, 5].

Medication management capacity (MMC) refers to the “cognitive and functional ability to comply with a medication regimen, when it is the individual’s wish or desire to follow a medication regimen as prescribed” [6]. MMC encompasses factors such as understanding the purpose and importance of medications, being able to remember and follow prescribed dosages and administration instructions and having the necessary skills to handle medication containers and administer medications correctly [6]. Medication management capacity is closely linked to adherence [7]. Medication adherence refers to “the extent to which a person’s medication‐taking behavior corresponds with agreed-upon treatment recommendations from a healthcare provider” [8, 9]. If an individual lacks the necessary cognitive or functional abilities to manage their medications effectively, it can result in unintentional nonadherence [6, 7, 9]. Compared to younger patient groups, concerns regarding medication management capacity are particularly significant among older adults [10]. According to the World Health Organization (WHO), approximately 46% of people over the age of 60 suffer from some form of disability, with visual impairment, hearing impairment, cognitive limitations, and osteoarthritis being the most common causes [11]. These limitations can impair the ability of older individuals to manage complex medications and, as a consequence, restrict their independence [11].

The MOLD-US framework, with its focus on physical, cognitive, sensory, and motivational barriers affecting the usability of mobile health applications in older adults, serves as a valuable guide for understanding and categorizing challenges in medication self-management [12]. By considering impairments associated with aging and their consequences, this framework addresses the challenges involved in medication management in older adults [12]. Physical impairments associated with aging include a decline in grip strength, dexterity, coordination, and mobility of the hands and arms [12]. Research on rheumatoid arthritis patients revealed that hand function deterioration associated with arthritis hindered their ability to open tablet containers and unit dose packs [13]. Aging also leads to a loss of certain cognitive abilities, including processing speed as well as certain memory, language, visuospatial, and executive functions [14, 15]. In addition, certain conditions, such as dementia, can worsen cognitive decline, which ultimately reduces medication management ability [14, 15]. Visual functions that decline with age include the ability to resolve detail, focus on close objects, discriminate between colors, detect contrast, adapt to darker conditions, and increase susceptibility to glare [16, 17]. A study involving 156 patients above the age of 65 compared issues related to self-management of medications among older individuals with and without visual impairment [16]. Despite using visual aids, approximately 29% of individuals with visual impairment required assistance managing their medications [16]. Moreover, age-related eye diseases such as cataracts, age-related macular degeneration, glaucoma and diabetic retinopathy can also deteriorate the vision functions of older individuals [17]. Motivational challenges that older adults encounter with medication self-management at home include inadequate knowledge about medications and the use of adherence technologies (health literacy and technology literacy), low self-efficacy, lack of confidence in taking medications properly, and integration of medication management during daily activities [12]. Additionally, research suggests that feedback from care partners and the environment in the home can impact the ability of older adults to self-administer medication [18, 19]. Therefore, when assessing various barriers to medication-taking, it is important to take into account a variety of environmental factors, including social factors such as support from family and caregivers and home environment [19, 20].

Several studies have emphasized the importance of assessing the functional ability of older adults to medication management in clinical practice as it serves as a guiding factor for planning, applying, and monitoring interventions aimed at optimizing medication management, allowing healthcare professionals to tailor strategies to individual needs and challenges [21–24]. However, despite the significance of this assessment, standardized evaluations of functional ability in medication management or medication self-management are not routinely performed in clinical settings [24]. Often, judgments regarding medication management ability rely on the clinician's intuition or reports provided by the patient or caregiver, which have limitations in terms of knowledge, insight, and objectivity [21]. Instruments that measure instrumental activities of daily living and medication adherence are sometimes used to assess medication management capacity, but they are insufficient for evaluating the specific skills required for independent medication management [21].

A number of instruments have been developed to assess an individual's functional and cognitive capacity to manage medications [22–25]. Drug Regimen Unassisted Grading Scale (DRUGS), Medication Management Instrument for Deficiencies in the Elderly (MedMaIDE), the Hopkins Medication Schedule (HMS), and the Medication Management Ability Assessment (MMAA) are the tools most recommended by various reviewers based on the medication management skills measured, administration time, scoring scale, type of medication regimen used, and psychometric properties [21–24]. It is important to note that while various tools exist, most are designed to identify cognitive and physical barriers to successful medication administration, and none are known to address all barriers to medication management. [21–23]. Furthermore, considering that motivational and environmental factors significantly influence an individual's medication-taking behavior, it is crucial to incorporate these factors when assessing medication management capacity [19, 26]. The integration of these diverse elements into a single tool enables healthcare professionals to acquire a comprehensive overview of an individual's medication management capacity. This comprehensive assessment facilitates targeted interventions that consider the interplay of physical, cognitive, sensory, motivational and environmental factors, potentially result in more effective support and strategies to enhance medication management.

This review aims 1) to identify tools that measure physical, cognitive, sensory, environmental, and motivational barriers to medication self-management in older adults, and 2) to understand the extent to which these tools assess various barriers Although previous reviews have been conducted, this review aims to include any new tools that have emerged since then and to consider a broader range of barriers, including physical, cognitive, sensory, motivational, and environmental factors. By synthesizing the existing evidence and offering a consolidated resource, we aim to assist healthcare professionals in selecting appropriate tools for assessing medication management capacity in older adults and contribute to the advancement of knowledge in this field.

## Methodology

This scoping review was informed by the guidance provided by the Arksey and O’Malley scoping study framework and the PRISMA Extension for Scoping Reviews checklist [27, 28]. Based on the direction from these two sources, the scoping review included the following stages: (1) identifying the research question, (2) identifying relevant studies, (3) study selection, (4) charting the data, and (5) summarizing and reporting the results.

### Stage 1. Identifying the research question

The research question was as follows: Which tools exist to measure physical, cognitive, sensory, environmental, and motivational barriers to medication taking in older adults?

For this study, we define "tools" as instruments, scales, or assessment methods specifically designed to measure, evaluate, or assess various factors, including physical abilities, cognitive functions, sensory capabilities, motivational factors, and environmental factors that can influence an older adult's capacity to manage medications.

The MOLD-US framework developed to evaluate barriers of older adults influencing usability of mobile health applications was used in this scoping review to guide the identification and categorization of barriers to medication taking in older adults [12]. Even though its primary purpose may differ, the framework allowed us to categorize the diverse barriers impacting older adults' medication self-management in a comprehensive manner as physical, cognitive, sensory, and motivational barriers. In addition to these barriers, we aimed to capture the broader contextual factors, including environmental factors such as social support and home environment (e.g., counter space, adequate lighting), that may influence medication-taking among older adults [18–20].

### Stage 2. Identifying relevant studies

Relevant articles were found by using a thorough search strategy consisting of both medical subject headings and keywords in 6 databases: PubMed (MEDLINE), Ovid Embase, Ovid International Pharmaceutical Abstracts, EBSCOhost CINAHL, APA PsycINFO, and Scopus. An experienced medical librarian (CC) constructed the database search strategies and conducted the search with input from the team. The search strategies contained synonyms for the following search concepts: medication, self-management, tools, functional impairment (e.g., impaired hearing, vision) and older adults. In each database, all keywords were limited to the title and abstract fields. All search strategy results were limited to the English language and the date range of 2002-2022. The final search strategies were run in each database on June 20th, 2022, and all results were exported to EndNote 20 (Clarivate Analytics, 20.2.1) for duplicate removal. Supplemental file 1 contains the full search strategy utilized in each database. After duplicate removal, the remaining results were exported into Covidence (Veritas Health Innovation, 2022) for screening.

### Stage 3. Study selection

Two team members (BB and HP) initially independently screened the titles and abstracts of 460 articles (10% of citations retrieved) based on the predetermined inclusion and exclusion criteria**.** The inter-rater reliability between the two researchers was determined (the Kappa coefficient was found to be 0.88). The remaining publications were screened by a single reviewer (BB) in view of this strong inter-rater reliability. Full-text screening of eligible studies was conducted by three team members (BB, AM, KP). One reviewer (BB) screened all the eligible studies, and the other two reviewers (AM, KP) screened 50% of the studies each. The bibliographies of the pertinent studies were also screened for additional relevant studies. Studies were included if they were (1) conducted in participants with a mean age of ≥60 years, (2) introduced or proposed tools designed to examine any of the physical, cognitive, motivational, and environmental barriers related to medication taking, or tools to assess functional decline/capacity/limitation/independence/disability related to medication-taking, (3) tools for which psychometric evaluation (at least one of reliability, content validity or construct validity) is available, (4) published between 2002 and 2022, (5) published in the English language, and (6) performed in the outpatient setting or after hospital discharge. The exclusion criteria were as follows: (1) studies performed in inpatients or assisted living residents, (2) editorials, comments, letters to the editor, guidelines, case series and case reports, (3) studies that reported on condition-specific tools (designed to be used in specific diseases only), (4) tools introduced to measure domains other than barriers to medication management, such as self-care or medication adherence, and (5) studies measuring physical, cognitive, sensory, motivational, and environmental barriers, but not related to medication-taking. Disagreements among the three reviewers were resolved through discussion and consensus. Where consensus was not achieved, a fourth team member (SA) was invited to assist with resolving the disagreement.

### Stage 4. Charting the data

Data abstraction from the included studies was completed using a Microsoft® Excel® (Office 365 Version 1906) spreadsheet. The following data were abstracted for each included study: title, author, year of publication, journal, country, age and gender of participants, sample size, study objective, study design, study duration, study setting, inclusion criteria, exclusion criteria, assessment tools mentioned, and main outcomes. For the identified tools, the following data were abstracted: purpose, administration time, type of instrument (performance-based/self-reported), type of medication regimen used, barriers assessed, and psychometric properties (validity & reliability). Two reviewers (BB, RS) abstracted data from eligible studies, and the accuracy of the abstracted data was verified by two additional reviewers (AM, KP).

### STAGE 5: Summarizing and reporting the results

The general characteristics of the studies and properties of the tools were collected and summarized. The results were then categorized and summarized based on the type of tool, barriers assessed, medication management skills assessed, and psychometric properties.

## Results

**Fig 1:**
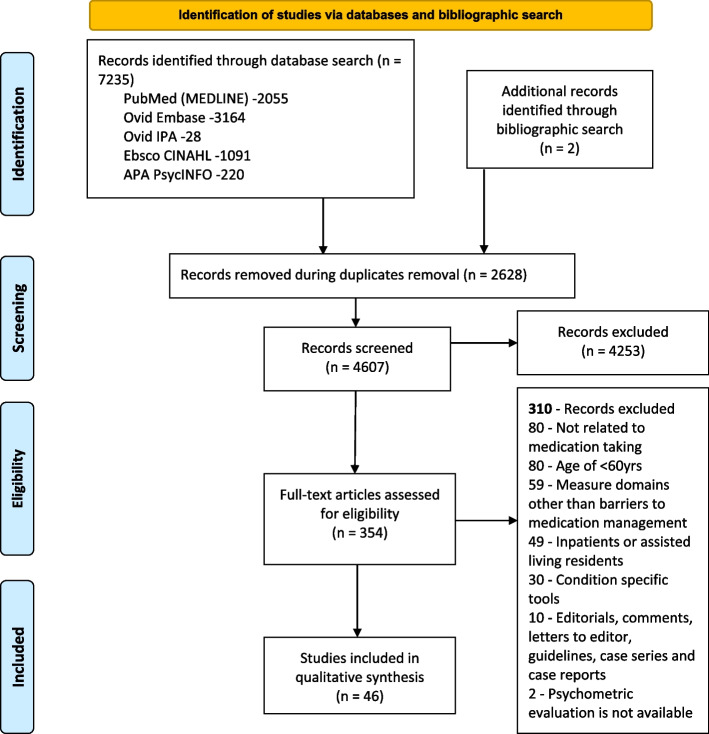
PRISMA flow diagram

A total of 7235 studies were identified. After removing duplicates, two reviewers screened 4607 articles by title and abstract, of which 4253 did not meet the inclusion criteria. Therefore, 354 articles were included for full-text review. Of these, 39 articles, four theses, one conference abstract, and two articles identified from the manual search of bibliographies met the inclusion criteria. In the 46 papers included, 44 tools measuring various barriers to medication management capacity were identified. The flow chart in Figure 1 illustrates the screening process.

### Study Characteristics

Publication rates varied across decades, with sixteen articles published from 2002 to 2012 and thirty from 2013 to 2022. More than half of the studies (*n*=25) were conducted in the United States, 13 in Europe, 4 in Asia, 2 in Australia, and 2 in Canada. A variety of study designs were used: cross-sectional (*n*=30), pilot study (*n*=5), cohort study (*n*=2), scoping review (*n*=2), validity study (*n*=3), case‒control study (*n*=1), mixed method study (*n*=1), systematic review (*n*=1), and randomized controlled trial (*n*=1). Most of the studies included both males and females, except for one study conducted on community-dwelling women aged 70 to 80 years. Twelve studies targeted older adults with specific conditions (coronary heart disease, Parkinson’s disease, chronic obstructive pulmonary disease, hypertension, age-related macular degeneration), one study recruited pharmacists and pharmacy students to evaluate the validity of a medication assessment tool for older adults, and the remaining studies targeted community-dwelling older adults. A detailed description of the studies included is summarized in Table 1.

**Table 1:** Study characteristics

**Author, Year of Publication, Country**	**Tool(s)**	**Study design**	**Study Objective**	**Population description**	**Sample Size**	**Mean age of Participants**	**Gender**	**Study outcome**
Advinha AM., et al., [29] 2021 Portugal	Drug Regimen Unassisted Grading Scale (DRUGS) Self-Medication Assessment Tool (SMAT)	Observational study (Cross sectional)	To assess the ability of older people to self-manage their medication.	Community-dwelling residents over 65 years old	207	75.5 years	Female (75.4%) Male (24.6%)	The probability that an older individual would be able to manage medications with total accuracy (100%) increases exponentially with cognitive competence. The functional ability of older people to self-manage medications was found to be clearly associated with cognitive impairment.
Caffery DM., et al., [30]2007 US	Cognitive Screen for Medication Self-Management (CSMS) Test in Older Adults	Validity study	To evaluate specific identified psychometric properties of the CSMS.	Community dwelling individuals age from 72 to 95 and living independently	60	NR	Female (75%) Male (25%)	Established validity for cognitive status and age, Reliability measure, Internal consistency, -0.08-0.84.
Insel, K., et al., [31] 2006 US	Mini-Mental State Examination (MMSE) Wisconsin Card Sorting Test (WCST) Digit Span Backward (DSB) California Verbal Learning Test (CVLT)	Observational study (cohort study)	To investigate the association between cognitive processes and medication adherence among community-dwelling older adults.	Older adults (67 years or older)	100	78 years	Female (78%) Male (22%)	Executive function and working memory tasks were the only significant predictor (b = .44, *p* < .01) of medication adherence. Assessments of executive function and working memory can be used to identify community-dwelling older adults who may be at risk for failure to take medicines as prescribed.
Kripalani, S., et al., [32] 2006 US	Drug Regimen Unassisted Grading Scale (DRUGS) Rapid Estimate of Adult Literacy in Medicine (REALM)	Observational study (Cross sectional study)	To evaluate the effects of low literacy, medication regimen complexity, and sociodemographic characteristics on MMC.	Patients with CHD	435	65.4 years	Female (54.6%) Male (45.4%)	Total DRUGS scores increased with literacy level (*P*=.001), as did the ability to identify medications correctly (*P*< .001). Patients with inadequate literacy specifically struggled with identifying their medications by viewing the bottle exterior or label (*P*< .001, compared with higher literacy patients).
Lam, AY., et al., [33] 2011 US	Mini-Cog Medi-Cog Medication-Transfer screen (MTS)	Observational study (Cross sectional study)	To assess pillbox, fill accuracy and cognition among community-dwelling older adults.	Community-dwelling older adults > 60 years of age,	50	76.4 years	Female (58%) Male (42%)	All components of the cognitive screens except the clock draw portion of the Mini-Cog were significantly associated with PC. The Mini-Cog and MTS individually accounted for about 30% of the variance (*P* < 0.001); their combination into the Medi-Cog was the strongest predictor of PC, accounting for 44% of the variance (*P* < 0.001). Medi-Cog was the strongest predictor of PC.
Lubinga, SJ., et al., [34] 2011 UK	Self-Medication Risk Assessment Tool (RAT)	Observational study (Cross sectional study)	To determine scale reliability and validate the instrument against community pharmacists' assessment of patients' ability to manage their medicines.	Older adults who were at least 65 years old	37	Median age-76years	Female (48.6%) Male (51.4%)	Cronbach’s alphas were 0.792, 0.679 and 0.813 for the 13-item, cognitive risk, and the physical risk sub-scales respectively. The total risk score and cognitive risk sub-scores were significantly worse among multi-compartment compliance aid users compared to the non-users.
Mortelmans, L., et al., [35] 2021 Belgium	Medication Management Instrument for Deficiencies in the Elderly (MedMaIDE)	Observational study (Cross sectional study	To describe post-discharge medication self-management by geriatric patients with polypharmacy, to describe the problems encountered and to determine the related factors.	Older adults aged least 75 years old, used five or more prescribed medicines.	400	82 years	Female (52.5%) Male (47.5%)	After discharge, 70% did fully self-manage their medication, 27% received help with preparing their medication but self-administered their medicines and 3% received help with preparing and administering medicines at home. Approximately 90% of patients experienced at least one medication management deficiency after discharge. Most deficiencies were related to medication knowledge (mean 3.1 [SD 1.8]).
Kim, JS., et al., [36] 2013 South Korea	Mini-Mental State Examination (MMSE) Montreal Cognitive Assessment (MoCA)	Observational study (Cross sectional study	To evaluate the correlation between ability to manage medication and cognitive functioning in patients with PD.	PD patients	208	66.4 years	Female (55.29%) Male (44.71%)	Correlations between PillQ scores and scores on the MMSE and MoCA approached moderate strength. Among the MMSE subscales, orientation (-0.403 *p*<0.001) and memory registration (-0.314 *p*<0.001) were most strongly related to scores on the PillQ. The orientation (-0.363 *p*<0.001) and visuospatial subscales (-0.375 *p*<0.001) of the MoCA were strongly correlated with PillQ scores.
Anderson, RE., et al., [37] 2016 US	Short Blessed Test (SBT) Montreal Cognitive Assessment (MoCA) Trail-Making Test (TMT)	Observational study (prospective)	To determine whether cognitive dysfunction, in particular impaired executive function, may be a risk factor for early readmission in older adults independently managing their medications.	Individuals aged 65 and older	452	74.7 years	Female (59.1%) Male (40.9%)	For participants managing medications themselves, adjusted 30-day odds of readmission increased 13% on average with each point decrease in SBT score (*P* = .003) and 9% on average with each 0.01 decrease in TMT-B score (*P* = .02).
Risser, J., et al., [38] 2007 US	Self-Efficacy for Appropriate Medication Use Scale (SEAMS) Rapid Estimate of Adult Literacy in Medicine (REALM)	Experimental study	To develop a self-efficacy scale for medication adherence in chronic disease management that can be used in patients with a broad range of literacy skills.	Patients with documented coronary heart disease (CHD) who presented to the clinic	436	63.8 years	Female (55.7%) Male (44.3 %)	The final 13-item scale had good internal consistency reliability (Cronbach’s α 0.89). Test-retest reliability of the 21-item scale was moderate (Spearman’s ρ 0.62, p 0.0001). Self-efficacy as measured by the scale was strongly correlated with medication adherence as assessed by the Morisky scale (Spearman’s ρ 0.51, p .0001).
Castel-Branco, M., et al., [39] 2015 Portugal	Mini-Mental State Examination (MMSE)	Observational study (Cross sectional study	To identify the elements required for an appropriate medication self-management in elderly in order to create a Good Practice Guideline for home visits to isolated polypharmacy elderly.	Patients with 65 or more years old, living alone	34	NR	NR	From a total of 37 seniors, only 62 % were considered non-adherents although 87 % knew when to take their medication, and 85 % reported using different memory strategies, such as associating the administration with a specific activity, pillboxes, or the location of the medicine at home.
Marks, TS., et al., [40] 2020 US	Mini-Cog Medi-Cog-R Medication-transfer screen-revised (MTS-R)	Observational study (Cross sectional study	To examine whether a combined cognitive and performance-based medication management measure would be able to better classify an individual’s functional cognitive status and potential for instrumental activities of daily living (IADL) impairment than either measure alone.	Community-dwelling adults	185	70.68 years	Female (76.2%) Male (23.8 %)	The Mini-Cog, the MTS-R, and the Medi-Cog-R all show discriminant validity, but the combined measure demonstrates greater sensitivity and specificity than either component measure alone in identifying IADL impairment.
O'Conor, R., et al., [41] 2019 US	Short Test of Functional Health Literacy in Adults (S-TOFHLA) Trail Making Test (TMT) Mini Mental State Exam (MMSE)	Observation study (cohort study)	To assess the association between health literacy and cognitive abilities with self -management behaviors in patients with COPD.	Adults with COPD	388	68 years	Female (58.3%) Male (41.7 %)	Compared with individuals with adequate health literacy, participants with limited health literacy were less likely to be adherent to their COPD medicines (23.3% vs. 46.0%, *p* < 0.001), demonstrate correct MDI (57.8% vs. 71.9%, p = 0.02) or DPI (40.0% vs. 56.7%, *p* = 0.04) technique, or have one healthcare provider regularly manage their COPD. Global cognitive ability was predictive of correct MDI and DPI technique.
Son, YJ., et al., [42] 2017 South Korea	Self‐Efficacy for Appropriate Medication Use Scale (SEAMS)	Observational study (Cross sectional study	To examine the mediating role of self‐efficacy in the relationship between depression and medication adherence among older patients with hypertension.	Older adults patients with hypertension	255	73.89 years	Female (48.2%) Male (51.8%)	Depression was significantly negatively correlated with self‐efficacy (r = −.26, P < .001) and medication adherence (r = −.24, *P* < .001), while self‐efficacy was significantly positively correlated with medication adherence (r = .53, P < .001), depression significantly predicted self‐efficacy (β = .20, P = .002) and medication adherence (β = −.28, *P* < .001).
Wajda, B., et al., [43] 2014 US	National Eye Institute Visual Function Questionnaire-25 (NEI VFQ–25)	Observational study (Cross sectional study-prospective)	To determine whether personality traits influence self-reported functional vision in patients with age-related macular degeneration (AMD).	Patients with AMD	182	84.1 years	Male (29%) Female (71%)	For near functional vision, visual acuity [95% confidence interval {CI} 0.46, 0.20]; p 0.001), and education [95% CI 0.01, 0.15]; p 0.03) were statistically significant predictors. For distance functional vision, only visual acuity [95% CI – 0.69, – 0.29]; p 0.001) was statistically significant predictor.
Yang, C., et al., [44] 2021 China	Medication-Specific Social Support Questionnaire (MSSS) The Self-Efficacy for Appropriate Medication Use Scale (SEAMS)	Protocol for a randomised controlled trial	To implement an evidence-based, theory-informed, and nurse-led medication self-management intervention among older patients with multimorbidity and examine its effects in community settings.	Community-dwelling older patients with multimorbidity.	NR	NR	NR	NR
Smith, SG., et al., [45] 2015 US	The Rapid Estimate of Adult Learning in Medicine (REALM) Test of Functional Health Literacy in Adults (TOFHLA-R) The Newest Vital Sign (NVS) Comprehensive Health Activities Scale (CHAS)	Observational study (Cross sectional study	To investigate the relationship between literacy and numeracy and their association with health task performance.	English-speaking adults ages 55 to 74	304	63.2 years	Female (74.7%) Male (25.3%)	Literacy and numeracy were both significantly associated with performance on all tasks (literacy range, b = 0.23–0.45, all ps < 0.001; numeracy range, b = 0.31– 0.41, all ps < 0.001).
Curtis, LM., et al., [46] 2016 US	The Rapid Estimate of Adult Learning in Medicine (REALM) Test of Functional Health Literacy in Adults (TOFHLA-R) The Newest Vital Sign (NVS) Comprehensive Health Activities Scale (CHAS)	Observational study (Cross sectional study)	To determine the prevalence of various forms of cognitive decline over a 3-year period, and to examine associations with requisite health literacy and self-management skills.	English-speaking adults ages 55 to 74	545	66 years	Female (69%) Male (31%)	Decline in long term memory was associated with poorer self-management skills (beta -3.26, 95%CI -4.96, -1.55; p < 0.001). Cognitive decline was not associated with performance on the REALM or the NVS assessments.
Sluggett, JK., et al., [15] 2020 Australia	Drug Regimen Unassisted Grading Scale (DRUGS) Self-Efficacy for Appropriate Medication use Scale (SEAMS)	Non-randomized pilot and feasibility study	To determine the feasibility of a multi-component intervention to simplify medication regimens for people receiving community-based home care services.	Older adults	25	79 years	Female (64%) Male (36%)	The DRUGS assessment showed most participants were able to self-manage their medications, participants who received intervention did so with a high degree of protocol adherence and acceptability. Simplification was possible for 14 participants (56%) and implemented for 7 (50%) at follow-up.
Beckman, A., et al., [47] 2005 Sweden	Mini-Mental State Examination (MMSE)	Observational study (Cross sectional study)	To investigate elderly people’s ability to open medicine containers, and how this ability correlates to some common disorders that may cause functional or cognitive impairment.	Older adults aged 75 years or older,	604	86.7 years	Male (22.4%) Female (77.6%)	14% were unable to open a screw cap bottle, 32% a bottle with a snap lid, and 10% a blister pack. Female gender, higher age, living in an institution, Parkinson’s disease, rheumatoid arthritis, cognitive impairment and impaired vision were all associated with a decreased ability to open the containers.
Somerville, E., et al., [20] 2019 US	HOME–Rx-revised Medication Management Instrument for Deficiencies in the Elderly (MedMaIDE) Medication Management subscale of the Performance Assessment of Self-care Skills (PASS)	Observational study (Cross sectional study)	To further develop the HOME–Rx, an in-home medication management assessment, by modifying scoring metrics, improving clinical utility, and establishing psychometric properties.	Older adults	Phase 1: 4 Phase2:30	Phase1-73.8 years Phase2-75.8 years	Phase1- Female (50%) Male (50%) Phase2- Female (73.3%) Male (26.7%)	Phase 1- Administration time was reduced from an average of 65 to 75 min to 25 to 35 min. Phase2: The PASS was positively correlated with the HOME–Rx Performance and Safety subscales; the MedMaIDE was negatively correlated with the HOME–Rx Performance subscale and positively correlated with the Barriers subscale. Interrater reliability was excellent (ICCs = .87–1.00).
Murphy, MC., et al., [48] 2017 US	In-Home Medication Performance Evaluation (HOME–Rx)	Validity study	To develop a novel, performance-based medication adherence assessment.	Older adult	12 Content expert participants 7 Older adult 5	75.6 years	Female (60%) Male (40%)	Content experts were in agreement that the overall instrument was valid for measuring medication management (scale-level CVI 5 .95). Older adult participants reported the instrument was relevant, acceptable, and easy to understand.
Hutchison, LC., et al., [49] 2006 US	Medication Management Ability Assessment (MMAA) Drug Regimen Unassisted Grading Scale (DRUGS) Mini-Mental State Examination (MMSE)	Observational study (Cross sectional study)	To compare the Medication Management Ability Assessment (MMAA) and the Drug Regimen Unassisted Grading Scale (DRUGS) as standardized tools to assess medication management skills in elderly patients with a range of cognitive function.	Individuals with Alzheimer's disease and a control group	52	75.8 years	Female (69%) Male (31%)	The 49 participants who took the MMAA had a mean (SD) score of 19.4 (6.1), with a range of 0 to 25. The 46 participants who took the DRUGS had a mean (SD) score of 91.6 (24.7), with a range of 0 to 100.The MMAA and the DRUGS correlated with one another (P = 0.000).
Miller, DJ., et al., [50] 2022 US	The National Eye Institute Visual Function Questionnaire (NEI-VFQ-25) Functional Health Literacy Scale (FHL)	Pilot study Prospective, single-arm pilot study with a pre post design.	To investigate whether demographic, clinical, or psychosocial factors act as moderators of change in medication adherence in the Support, Educate, empower (SEE) program.	Glaucoma patients	39	63.9 years	Female (44%) Male (56%)	There were no significant differences in the slopes of adherence for better-eye MD, visual acuity, number of comorbidities, visual function measured by the NEI-VFQ-25 score, FHL or GMSE in response to medication reminders (P > 0.05) for all comparisons.
Advinha, AM., et al., [22] 2016 Portugal	Self-Medication Assessment Tool (SMAT)	Pilot study	To assess elderly’s medication management ability using the Self Medication Assessment Tool – Portuguese Version (SMAT-PT) and to correlate the performance between standard and real therapeutic regimens.	Portuguese community-dwelling elders	150	74.73 years	Female (74.7%) Male (25.3%)	The SMAT-PT standard regimen mean scores were 20.92 (±6.83) in functional ability and 38.75 (±5.92) in cognitive ability. Significant correlations between medication recall and standard regimen items were found. Cognitive measures were directly correlated with medication management ability.
Alosco, ML., et al., [51] 2012 US	Mini Mental State Examination (MMSE) Trail Making Test (TMT)	Observational study (Retrospective observational analyses)	To examine whether cognitive functioning predicts instrumental ADL performance in persons with HF.	HF population	122	68.49 years	Female (35.2%) Male (64.8%)	In each case, poorer neuropsychological test performance was associated with poorer instrumental ADL function. Poorer cognitive test basic performance was associated with reduced independence in medication management
Bailey, S., et al., [52] 2015 US	Measure of Drug Self-Management (MeDS)	Observational study (Cross sectional study)	To develop and evaluate a comprehensive yet brief Measure of Drug Self-Management (MeDS) for use in research and clinical settings among diverse patient groups.	Diagnoses of diabetes and hypertension	193	61.1 years	Female (60.1%) Male (39.9%)	MeDS demonstrated adequate internal consistency with a Cronbach’s α of 0.72. The scale was significantly correlated with the Morisky Medication Adherence Scale (r= -0.62; P,0.001), low-density lipoprotein cholesterol (r= -0.27, P<0.001) and diastolic blood pressure (r= -0.18, P=0.01).
McCann, RM., et al., [16] 2012 Australia	Daily Living Tasks associated with Vision (DLTV)	Observational study (case–control study)	To compare issues relating to medication self-management between older people with and without VI.	Individuals aged ≥65 years,	Visually impaired-156, Control-158	Visually impaired-81 years Control-77.8 years	Visually impaired- Male (35.9%) Female (64.1%) Control-\ Male-(38.6 %) Female -(61.4 %)	Significantly more with VI (29%), compared to controls (13%) (OR = 2.8 [95% CI = 1.6 to 5.0]; age-adjusted OR = 2.6 [95% CI = 1.4 to 4.7]) relied on help to take their medication each day or to sort it into a compliance aid (a container holding usually seven daily aliquots of medication, each within separate sections).
Raehl, CL., et al., [53] 2002 US	Med Take test Whisper test Mini-Mental State Examination (MMSE)	Observational study (Cross sectional study)	To quantify how seniors’ ability to take oral prescription drugs safely may correlate with age, sex, socioeconomic status, education, cognitive impairment, depression, and drug self-management.	Older adults	57	79.49 years	Female (72%) Male (28%)	Significant predictors of the outcome MedTake test score, adjusted for age and sex, were MMSE (b = 0.393, p=0.002) and Medicaid assistance in last 10 years (b = -0.302, p=0.021).
Creech, CL., et al., [54] 2016 US	Self-Efficacy for Appropriate Medication use Scale (SEAMS) Newest Vital Sign (NVS)	Pilot study	To determine whether a brief, low-HL tailored intervention on common medication management issues could affect immediate changes in the dependent variables of knowledge and self-efficacy (SE).	Independently living older adults (Greater than 65 years)	14	84.06 years	Female (92.8 %) Male (7.2 %)	Post-test knowledge scores were significantly higher than pre-test scores for all participants (M = 8.43, Mdn = 9.00, SD =1.651 versus M = 3.93, Mdn = 4.00, SD = 1.817; p < .001). Change in knowledge and SE scores were not related to age, educational attainment, or baseline HL status.
Chin, J., et al., [55] 2021 US	Rapid Estimate of Adult Literacy in Medicine (REALM)	Observational study (Cross sectional study)	To examine how health literacy and its components (processing capacity and knowledge about illness) influence memory for medication purposes.	Individuals with diagnosis of type II diabetes mellitus	674	63.6 years	Female (55.2%) Male (44.8 %)	Health literacy was associated with memory for medication purposes, with processing capacity and health knowledge partly mediating this association. (F (5,665) = 18.97, p < .001, adjusted R2 = 0.12, SE = 0.94).
Sumida, CA. [56],et al., 2019 US	Medication Management Ability Assessment (MMAA)	Observational study (Cross sectional study)	To examine the performance of healthy older adults’ (HOA) and individuals with amnestic mild cognitive impairment (aMCI) on the medication management abilities assessment’s original scoring criteria and derived error process measures.	Healthy older adults and individuals with amnestic mild cognitive impairment (aMCI)	50 Healthy older adults-25 Individuals with aMCI- 25	HOAs- 70.68 aMCI- 70.80	HOAs- Female-(68%) Male (32%) aMCI- Female (80%) Male (20%)	Individuals with aMCI performed more poorly than HOAs on the MMAA score and process error measures. The aMCI group showed significantly poorer performance on measures of total overtaking error (η2 = .169), total undertaking error (η2 = .099), the magnitude error score (η2 = .291) and the MMAA score (η2 = .258).
Thuy LT., et al., [57] 2020 Thailand	Multidimensional Scale of Perceived Social Support (MSPSS) Short Test of Functional Health Literacy in Adults (S-TOFHLA)	Observational study (Cross sectional study)	To examine the factors of medication regimen complexity, physical function, social support, health literacy, patient-provider communication, health belief, and self-efficacy in explaining medication adherence of older people with hypertension.	Individuals aged 60 years or older; being diagnosed with HTN and undertaking antihypertensive drug for at least 6 months;	300	68.11 years	Female (42%) Male (58%)	Five variables (medication regimen complexity, health literacy, patient-provider communication, health belief, and self-efficacy) were significantly associated with medication adherence. Physical function and social support were not significantly related to medication adherence (-.136*, -.114*).
Windham, BG., et al., [58] 2005 UK	Hopkins Medication Schedule (HMS) Pelli-Robson letter sensitivity chart (PR test) Randot Circles (stereovision) Early Treatment Diabetic Retinopathy Study eye chart (ETDRS)	Observational study (Cross sectional study)	To assess relationships between vision (Contrast sensitivity, stereopsis, visual acuity) and a performance-based measure of ability to implement new medications.	Community-dwelling women aged 70 to 80 years	335	76.8 years	only female	Each vision measure was positively associated with Pillbox Ratio scores and varied with cognition and time to completion. Better visual acuity, contrast sensitivity, and stereopsis were each associated with better performance in women with poor cognition who filled the pillbox quickly.
Robnett, RH., et al., [59] 2007 US	ManageMed Screening (MMS) Hopkins Medication Schedule(HMS)	Observational study (Cross sectional study)	To introduce ManageMed and complete initial reliability and validity analyses on the ManageMed Screening.	Volunteer participants, aged 65 and over.	67	76 years	NR	Adequate reliability and concurrent validity were established. Internal consistency, Cronbach’s Alpha of 0.89 (42 items). Interrater reliability on individual questions ranging from 0.859 to 0.965. A moderate correlation was attained between ManageMed total score and the total Cognistat score (0.696, p = 0.01), indicating that the results for both tests are similar (concurrent validity).
Russell, AM., et al., [60] 2018 UK	Rapid Estimate of Adult Literacy in Medicine (REALM)	Observational study (Cross sectional study)	To explores patient preferences for functionality in a smartphone application (app) that supports medication self-management among older adults with multiple chronic conditions.	English-speaking older adults (55 and older) who owned smartphones and took five or more prescription medicines	46	65 years	Female-70% Male-30%	Desired features included (1) a list and consolidated schedule of medications, (2) identification and warning of unsafe medication interactions, (3) reminder alerts to take medicine, and (4) the ability record when medications were taken.
Irvine-Meek, J., et al., [61] 2010 Canada	Self-Medication Assessment Tool (SMAT)	Observational study (Cross sectional study)	To evaluate the face validity of the SMAT and to determine its acceptability among pharmacists.	Pharmacists and pharmacy students	20	NR	NR	Participants rating the SMAT; 70% (14/20) for usefulness, 35% (7/20) for ease of use, 60% (12/20) for thoroughness, and 55% (11/20) for willingness to use. Pharmacists and pharmacy students working in hospital settings were more willing to use the SMAT than those working in community settings (p = 0.08, effect size = 0.17).
Haus, CS., et al., [14] 2003 US	Mini-Mental State Exam (MMSE) Martin and Park Environmental Demands Questionnaire (MPED) Long-Term Medication Behavior Self-Efficacy Scale (LTMBSES) Perceived Social Support from Friends (PSS-Fr) and the Perceived Social Support from Family (PSS-Fa)	Observational study non-experimental descriptive-correlational research design	To describe factors and medication strategies used by community dwelling elderly persons who live alone.	Older adults living alone	60	77.4 years	Females (90%) Males (10%)	No significant association was found between the outcome and the 7 predictor variables (MMSE, GDS-S, SS-Fa, SS-Fr, MSE, MPED-routine, MPED-busyness) (Wilks’ lambda is .822 (x2 = 10.637; p = .154))
Visscher BB., et al., [62] 2020 Netherland	Functional, Communicative and Critical Health Literacy scales (FCCHL)	Observational study Two-phase qualitative study	To explore the needs of people with low health literacy and DM2 regarding medication self-management and to explore the preferences for medication self-management support.	People with DM2 and low health literacy	18	NR	Female- (39%) Male- (61%)	The participants preferred to be supported with reliable and easily understandable Information, adequate interactive communication with health care professionals and fellow people with diabetes and tools for medication self-management support.
Klymko , KW., et al., [63] 2008 US	Fuld Object-Memory Evaluation (FOME)	Pilot study	To examine the prevalence of selected cognitive impairments and explore the relationships among cognitive function, hypertension related self-care, and blood pressure in African American older adults.	African American men and women aged 60 and older	39	70 years	Female (69%) Male (31%)	46% African American elders had a high prevalence of cognitive impairments. A strong positive association was found between cognition(memory) and HTN related self-care (correct medication use) (r=0.59 p<0.05).
Westerbotn, M., et al., [64] 2008 Sweden	Mini-Mental State Examination (MMSE)	Descriptive study	To describe how older people living at home experienced the management of their own medication regimen from their own perspective.	Individuals aged ≥85 years, living at home	25	89.8 years	Female (64%) Male (36%)	Most participants managed their medicines by themselves and were very content with this. Most important components for older people were to have good cognitive ability, to be independent and to get support with their medicines from a close person as a backup.
Deupree JP, et al., [65] 2011 UK	Test of Functional Health Literacy in Adults (TOFHLA-R) Medication Administration Self-Efficacy Scale (MASES)	Mixed method study	To explore how community dwelling adults ages 60 to 74 self-manage five or more daily prescription medications.	Community dwelling older adult	15	71.27 years	Female (87%) Male (13%)	Regardless of the health literacy level or the number of daily prescribed medications, participants demonstrated high accuracy of self-management for their medications.
Kapoor A., et al., [66] 2018 UK	Show back	Observational study (Cross sectional study)	To develop and test a comprehensive simulation which assesses older adult medication self-management proficiency.	English-speaking individuals aged 65+	9	76 years	NR	Inter-rater agreement- high proficiency across all five domains (83%–100%).

### Tool properties

Among the 44 tools, two broad categories were identified: performance-based (*n*=30) and self-report measures (*n*=14). Performance-based measures involved asking older adults to complete different tasks related to medication management or different instrumental activity tasks, while self-reported measures are based on subjective information provided by individuals as part of surveys and offer insights into aspects of their own lives that are not directly observable. Of the included tools, 19 measured a combination of various barriers, while others assessed only cognition (*n*=12), vision (*n*=5), motivational (*n*=4), environmental (social support) (*n*=3), or auditory (*n*=1) factors.

A detailed description of the tools identified is summarized in Tables 2 and Table 3 illustrates the type and extent of barriers assessed by these tools.

**Table 2:** Tool properties

Tools	Purpose	Number of Items	Scoring scale	Administration time	Type of instrument	Type of Medication regimen used	Medication management skill assessed	Psychometric properties -Study	Validity		Reliability		
									Content	Construct	Inter-rater	Test-retest	Internal consistency
**Physical + Cognition + Sensory + Motivation**													
ManageMed Screening (MMS) [59]	To quickly determine if someone can handle a moderately difficult medication routine	33 item	0-39	15-20 minutes	Performance-based	Simulated medication regimen	Read Rx label, recall information, open/close vials, perform calculations, organize pillbox	[59]	+	Neurocognitive function (Cognistat) (Pearson Correlation Coefficient of .696)	(0.86- 0.96)		0.89
Self-Medication Risk Assessment Tool (RAT) [34]	To assess elderly patients' needs for additional support in managing their medicines	13 item	0-26	5-20 minutes	Performance-based	Simulated and patient’s medication regimens	Read Rx labels, open different medication packaging, manipulate with 5 ml spoon and eye or ear drop bottles	[67]	+	Patient’s comprehension and dexterity of handling the medications			(≥0.79)
Cognitive Screen for Medication Self-Management (CSMS) [30]	To assess the sensory and cognitive constructs associated with medication adherence	8 item	15	NR	Performance-based	Simulated medication regimen	Bottle opening, label reading, clock reading, dose calculations, arrangement time, study time, immediate recall, delayed, recall, cued recall, prospective memory and dose planning	[30]	+	Cognitive status and age			-0.08-0.84
**Physical + Cognition + Sensory + Environmental**													
Medication Management Ability Assessment (MMAA) [48, 56]	To assess geriatric mental health patients’ ability to independently manage medications	4 item	0-25	45-60 minutes	Performance-based	Simulated medication regimen	Recall information, describe full regimen, open/close, remove the dose from vials, differentiate tablet by color	[68]	+	Cognitive function (neuropsychological l battery test) Adherence		0.96	
Self-Medication Assessment Tool (SMAT) [22, 29, 61]	To screen for medication self-management deficits in older adults and to facilitate targeted interventions	44 item	Multiple scale	45-60 minutes	Performance-based	Patient's own medication regimen Simulated medication regimen	Read Rx labels, recall information, interpret medication instructions, open vials, remove tablets form packaging, differentiate tablets by color, organize pillbox	[69]	+	Cognitive function (MMSE, CDT, CCT), Medication regimen complexity, Self-reported adherence	+(≥0.79)	+(≥0.83)	+(≥0.81)
**Physical + Cognition + Motivation + Environmental**													
HOME-Rx revised [20]	To assess ability to manage medication routines in context, identify risk factors for medication management problems, and identify the environmental barriers influencing medication management ability	4 subscales	Multiple scale	25 to 35 minutes	Performance-based	Patient's own medication regimen	Knowledge of medications, Recall information, maniple of medication bottles and/or syringe, and calculate medication doses, storing and retrieving pills; reading labels; verbalizing the dosage instructions, special instructions, and purpose; following dosing directions correctly and recognizing when one has missed doses; opening containers; setting up medications; taking out medications; and physically administering medications.	[20]	+	PASS (positively correlated with the HOME–Rx Performance subscale (r = .57, p < .001) and Safety subscale (r = .49, p < .001)) MedMaIDE (negatively correlated with the HOME–Rx Performance subscale (r = −.69, p < .001) and positively correlated with the HOME–Rx Barriers subscale (r = .70, p < .001)) , I–HOPE Assist			.87 to 1.00
Medication Management Instrument for Deficiencies in the Elderly (MedMaIDE) [20, 35]	To identify the deficiencies in older adults’ ability to take their medication at home.	20 item	0-13	30 minutes	Performance-based	Patient's own medication regimen	Medication knowledge (name all drugs and describe full regimen including indication, rout of administration, dose and time), Medication taking ability (filling a glass of water, sip enough water, open bottles/vials, remove dose from package, and demonstrate admiration method for oral and non-oral dosage form),Knowledge about ongoing supplies (identify existing refills, name of pharmacy or physician office, and available resources)	[70]	+	Cognitive function (MMSE) Functional status (ADL) Med. adherence (pill count)	0.74	0.93	0.71
**Physical + Cognition+ Motivation**													
Show Back [66]	To assess older adult medication self-management proficiency	5 item	0-100	22 minutes	Performance-based	Simulated medication regimen	Identify medications, explaining the indication, organizing pillbox, describing the administration process for injectables and inhaled medications, describing the timing of doses	[66]	+	Medication Discrepancy Tool (MDT)	0.83-1		
MedTake test [53]	To quantify seniors’ ability to take oral drugs safely, standardize the brown bag review	4 item	0-100	30-45 minutes	Performance-based	Patient's own medication regimen	Identify meds & recall med names, open bottles/vials & remove dose from package, state indication, food/water congestion, and timing	[53]	+	Cognitive function (MMSE) Educational level			
HOME-Rx [48]	To assess an older adult’s ability to manage medication routines in the home and to identify at-risk behaviors by home health occupational therapists	16 item	1-16	30-45 minutes	Performance-based	Patient's own medication regimen	Knowledge of medications, recall information, maniple of medication bottles and/or syringe, and calculate medication doses	[48]	+	Cognitive function (MoCA) MMC (MangeMed)			.87 to 1.00
Hopkins Medication Schedule (HMS) [58, 59]	To test older adults’ ability to understand and implement a routine prescription medication	2 item	0-11	15-30 minutes	Performance-based	Simulated medication regimen	Read Rx labels, comprehend medication regimen, plan a schedule for meds regimen, open & close vails, remove dose from vials, organize pillbox.	[71]	+	Cognitive function (MMSE) Functional status (IADL)		0.38	
**Cognition + Sensory + Motivation**													
Performance Assessment of Self-care Skills (PASS-IADL) [48]	To measure occupational performance of daily life tasks	26 (four domains)	NR	1.5-3 hour	Performance-based	NA	NA	[72]	+		0.29-0.43	0.82-0.97	0.94-0.96
**Physical + Cognition**													
Drug Regimen Unassisted Grading Scale (DRUGS) [15, 29, 49, 59]	To assess Medication self-management ability	4 item	0-100	5-15 minutes	Performance-based	Patient's own medication regimen	Identification: showing the appropriate medications, access: opening the appropriate containers, dosage: dispensing the correct number per dose, and timing: demonstrating the appropriate timing of doses	[73]	+	Cognitive function (MMSE) Functional status (ADL & IADL) Self‐reported MMC Health literacy		0.83	0.81
**Cognition + Motivational**													
Short Test of Functional Health Literacy in Adults (S-TOFHLA) [41]	To measure patients’ ability to read and understand the things they commonly encounter in the health care setting using actual materials like pill bottles and appointment slips	4 Numeracy items and 2 prose passages	0-100	12 minutes	Performance-based	Patient's own medication regimen		[74]	+	REALM			0.68-0.97
Test of Functional Health Literacy in Adults (TOFHLA-R) [45, 46, 65]	To measure the functional health literacy of patients.	50-item reading comprehension and 17-item numerical ability test	0-50	22 minutes	Performance-based	Patient's own medication regimen	Reading comprehension and numeracy	[75]	+	REALM WRAT-R		0.92	0.98
Comprehensive Health Activities Scale (CHAS) [45, 46]	To measure health literacy skills	45 item	0-100	60 minutes	Performance-based	Simulated medication regimen	organizing and dosing medication	[76]	+	TOFHLA and the NVS, REALM and the MMSE		> 0.80	
Functional, communicative and critical health literacy scales (FCCHL) [62]	Three newly developed scales for measuring functional, communicative, and critical HL among patients with type 2 diabetes in order to propose a measure of HL	14 item	4-point Likert scale ranging from ‘‘never’’ (1) to ‘‘often’’ (4)	NR	Self-reported	Patient's own medication regimen	NR	[77]	+			0.67-0.72	0.87
**Motivation + Environmental**													
Long-Term Medication Behavior Self-Efficacy Scale (LTMBSES) [14]	To measures self-efficacy in relation to medication compliance	22 item	Multiple scales	NR	Self reported	NA	NA	[78]	+	Various levels of adherence			0.88
Self-efficacy for Appropriate Medication Use Scale (SEAMS) [15, 38, 42, 44, 54]	To assess self-efficacy for appropriate medication use	21 item	21-63	5-10 minutes	Self-reported	Patient's own medication regimen	NR	[15, 38, 42, 44, 54]	+	REALM Various disease Various literacy levels		0.62	0.90
**Cognition**													
Mini-Mental State Examination (MMSE) [31, 36, 39, 47, 49, 51, 53, 64]	To check for cognitive impairment (problems with thinking, communication, understanding and memory)	11 item	0-30	10 minutes	Performance-based	NA	Cognitive ability to manage medications	[79]	+	Mattis Dementia Rating Scale Wechsler Adult Intelligence Test, Functional Independence Measure, Montgomery Asberg Depression Rating Scale . Zung Depression Scale.	0.69	0.96	0.96
Wisconsin Card Sorting Test (WCST) [31]	To assess abstract reasoning ability and the ability to shift cognitive strategies in response to changing environ-mental contingencies and also considered a measure of the executive functions.	14 item		12–20 minutes	Performance-based	NA	Cognitive ability to manage medications	[80]	+				0.93
Digit Span Backward (DSB) [31]	To assess working memory	8 item	0-16	Less than 5 minutes	Performance-based	NA	Cognitive ability to manage medications	[81]	+	Wechsler Adult Intelligence Scale		0.76-0.95	
California Verbal Learning Test (CVLT) [31]	To assesses encoding, recall and recognition	16 item		30 minutes	Performance-based	NA	NA	[82]	+			0.80–0.84	
Mini-Cog [33, 40]	To evaluate cognition in older adults	4 item	0-5	3 minutes	Performance-based	Pillbox	Read Rx labels, interpret medication instructions, organize pillbox	[83]	+	Abbreviated mental test score (AMTS), the Geriatric Depression Scale	0.76	0.86	0.83
Medi-Cog [33, 40]	To assess patients’ ability to fill their own prescribed medications into a pillbox	3 item	0-10	7-8 minutes	Performance-based	Pillbox	Read Rx labels, interpret medication instructions, organize pillbox	[84]	+	Cognitive function Correctly filled pills			
Medication-Transfer Screen (MTS) [33, 40]	To assess patients’ ability to fill their own prescribed medications into a pillbox	4 item	5	5 minutes	Performance-based	Pillbox	Read Rx labels, interpret medication instructions, organize pillbox	[84]	+	Cognitive function Correctly filled pills			
Montreal Cognitive Assessment (MoCA) [37, 65]	It assesses different cognitive domains: attention and concentration, executive functions, memory, language, visuoconstructional skills, conceptual thinking, calculations, and orientation	30 item	0-30	10 minutes	Performance-based	NA	NA	[85]		Age, educational levels, economic status, and sex, MMSE		0.92	0.82
Short Blessed Test (SBT) [37]	This test addresses cognitive concerns in the areas of orientation, memory, and concentration.	6 item	0 – 28	5-10 minutes	Performance-based	NA	NA	[86]		MMSE			0.52-0.58
Trail-Making Test (TMT) [24, 37, 51]	To assess executive function	25 item	Part A- 1-39 sec Part B-1-91 sec	5-10 minutes	Performance-based	NA	NA	[87]		Category Test (CAT), Wisconsin Card Sort Test (WCST), Paced Auditory Serial Addition Task (PASAT), Visual Search and Attention Test (VSAT).		Part A-0.78 Part B- 0.67	
Measure of Drug Self Management (MeDS) [52]	An assessment of medication self-management skills	NR	0-12	NR	Self-reported	Patient's own medication regimen	NR	[52]	+	Morisky Medication Adherence Scale and relevant clinical measures (HbA1c, blood pressure, and low-density lipoprotein cholesterol)			0.72
Fuld Object-Memory Evaluation (FOME) [63]	To assess memory	10 item	0-10	15 minutes	Performance-based	NA	NA	[88]	+			0.71	0.84
**Sensory**													
National Eye Institute Visual Function Questionnaire -25 (NEI VFQ–25) [43, 50]	To measures the dimensions of self-reported vision-targeted health status that are most important for persons who have chronic eye diseases.	25+1 item	0-100(Multiple scale)	10 minutes	Self-reported	NA	NA	[89]	+	Various eye disease 51-item NEI VFQ			0.71-0.85
Daily Living Tasks associated with Vision (DLTV) [16]	To assess functional impairment among patients with age-related macular degeneration (AMD)	24 item	0-100	6-10 minutes	Self-reported	NA	NA	[90]	+				0.97
Pelli-Robson letter sensitivity chart (PR test) [58]	To measures a patient's contrast sensitivity (CS) by finding the lowest contrast letters he/she can read correctly	NR	NR	NR	Self-reported	NA	NA	[91]					
Randot Circles [58]	To test the patient depth perception along with normal stereo vision.	NR	NR	NR	Self-reported	NA	NA						
Early Treatment Diabetic Retinopathy Study eye chart (ETDRS) [58]	To measure visual acuity	5 letters of equal difficulty on each row, with standardized logarithmic spacing between letters and rows: a total of 14 lines (70 letters)	NR	NR	Performance-based	NA	NA	[91]		Accuracy-0.12±0.14		Test -retest variability-0.23±0.17	
Whisper test [53]	To assess hearing	6 steps	Threshold for hearing impairment <50% correct	5 minutes	Self-reported	NA	NA	[92]		Sensitivity (%; 95% CI) 100 (96-100) ,) Specificity (%; 95% CI) 87 (80-92			
**Motivation**													
The Newest Vital Sign (NVS) [46, 54, 61]	To identify patients at risk for low health literacy.	6 item	0-6	3 minutes	Performance-based	Patient's own medication regimen	Read Rx labels, interpret medication instructions	[93]	+	TOFHLA			0.76
Rapid Estimate of Adult Literacy in Medicine (REALM) [32, 35, 38, 45, 46, 55, 60]	To assess an adult patient's ability to read common medical words and lay terms for body parts and illnesses	7 item	0-66	2-3 minutes	Performance-based	NA	NA	[94]	+	Peabody Individual Achievement Test-Revised (PIAT-R) Wide Range Achievement Test-Revised (WRAT-R), Slosson Oral Reading Test-Revised (SORT-R)		0.99	0.97
Medication Administration Self-Efficacy Scale (MASES) [65]	To identify levels of self-efficacy, self-care, trust, levels of support from the community and organizations, and satisfaction levels related to self-administration of medications	26 item	0-3	NR	Self-reported	Patient's own medication regimen	NA	[65]	+				0.95
Martin and Park Environmental Demands Questionnaire (MPED) [14]	To measure two dimensions of environmental demand: (1) busyness and (2) routine	13-item	Likert scale 1 through 5.	5-10 minutes	Self-reported	NA	NA	[95]	+	Age Household size Medication-taking errors. (External validity)			0.88 for the busyness scale and 0.74 for the routine scale
**Environmental**													
Medication-Specific Social Support Questionnaire (MSSS) [44]	To identify how often participants received help for their medication taking over a three-month period	8 items	0-4	NR	Self-reported	NA	NA	[96]	+	Various diseases, drugs			0.92
Multidimensional Scale of Perceived Social Support (MSPSS) [57]	To assess an individual’s perception of the social support he or she receives from family, friends and significant others	12 item	7-point Likert type scale	5-10 minutes	Self-reported	NA	NA	[97]	+			0.91	0.95
Perceived Social Support from Friends (PSS-Fr) and the Perceived Social Support from Family (PSS-Fa) [14]	To measure the extent to which an individual perceives that his/her needs are fulfilled by friends and family	20 item	0-20	NR	Self-reported	NA	NA	[98]	+	Various symptoms of distress and psychopathology, mood states			0.88 for PSS-FR and 0.90 for PSS-FA

### Psychometric properties

There was at least one validity (content and construct) and one reliability (inter-rater, test-retest, internal consistency) data reported for most of the tools we reviewed. For MedTake, Medi-cog, and MTS, only validity data (both content and construct) were reported. Construct validity was shown through association with cognitive function and correctly filled pills for MTS and Medi-cog. The MedTake test was validated for construct validity using cognitive function (MMSE) and educational level. For the ETDRS eye chart, the psychometric properties were measured in terms of accuracy (-0.12*0.14) and test-retest variability (-0.23*0.17). Sensitivity - 100% (95% CI: 96-100) and specificity - 87% (95% CI: 80-92) were reported for the whisper test as psychometric measures. Table 2 contains a detailed description of the psychometric properties of each tool identified.

## Discussion

For older adults, managing multiple health conditions with complex medication regimens can be quite challenging, potentially affecting their quality of life [4]. Assessment and identification of specific limitations in medication management capacity can promote a deeper understanding amongst healthcare providers of how these challenges influence adherence to treatment as well as implementation of appropriate strategies to mitigate the impact on adherence [22–25, 29, 49, 56, 99]. We aimed to identify a tool that comprehensively evaluates various barriers to medication self-management, including physical, cognitive, sensory, motivational, and environmental domains. Although we found 44 tools that assess these barriers either separately or together, no single tool collectively addressed all five barriers.

### Assessment domains and promising tools

There are significant differences in the type and extent to which physical, cognitive, sensory, motivational, and environmental barriers are assessed in the tools we identified. While there are several instruments that exist to measure various aspects of physical and cognitive barriers, sensory components such as color vision, dark adaptation, and auditory factors, along with socio-economic factors including cost considerations and the home environment, are less frequently or thoroughly addressed. Instruments such as the Self-medication Assessment Tool (SMAT), ManageMed Screening (MMS), Self-medication Risk Assessment Tool (RAT), HOME-Rx revised, Medication Management Ability Assessment (MMAA), Medication Management Instrument for Deficiencies in the Elderly (MedMaIDE), and MedTake test stand out for their degree of assessment, each assessing between 11 to 16 of the 29 components [20, 29, 34, 35, 49, 56, 59, 61, 99]. However, it is important to highlight that the tools predominantly assess physical and cognitive domains. Previous studies by Farris and Phillips, Elliot and Marriott, and Badawoud et al. have also confirmed the effectiveness of tools like DRUGS, MedMaIDE, MedTake test, MMAA, and HMS in determining physical and cognitive abilities for independent medication management [21, 23, 24].

This focus on physical and cognitive barriers underscores a significant gap in the assessment of other critical domains, especially sensory and socio-economic factors. Sensory components, such as visual and auditory factors, are essential for accurately identifying and managing medications, yet they are often not considered in current assessment tools. Socio-economic factors, including affordability and the suitability of the home environment for medication management, also play a significant role in an individual's ability to adhere to medication regimens but are similarly under addressed. The limited emphasis given to sensory, motivational and environmental barriers highlights the necessity for further research.

**Table 3:** Tools and type of barriers assessed

Tools	Physical					Cognitive						Sensory			
	Speed of performance	Flexibility of joints	Hand - eye coordination	Retention in hand movement	Grip strength	Working Memory	Spatial cognition	Dynamic /selective attention	Phonemic/semantic fluency	Reasoning	Numeracy and representational fluency	Vision	Visual acuity/accommodation	Color Vision	Contrast detection
MMS		✓	✓	✓	✓	✓	✓	✓	✓	✓	✓	✓		✓	
RAT		✓	✓	✓	✓	✓	✓	✓		✓	✓	✓	✓		
CSMS	✓	✓	✓	✓	✓	✓	✓	✓	✓	✓	✓	✓	✓	✓	
MMAA	✓	✓	✓	✓	✓	✓	✓	✓		✓	✓			✓	
SMAT	✓	✓	✓	✓	✓	✓	✓	✓	✓	✓	✓	✓	✓	✓	✓
HOME - Rx revised		✓	✓	✓	✓	✓	✓	✓	✓	✓	✓				
MedMaIDE		✓	✓	✓	✓	✓	✓	✓		✓	✓				
Show Back		✓	✓	✓	✓	✓	✓	✓							
MedTake test	✓	✓	✓	✓	✓	✓	✓	✓		✓	✓				
HOME - Rx		✓	✓	✓	✓	✓	✓	✓	✓	✓	✓				
HMS	✓	✓	✓	✓	✓	✓	✓	✓		✓	✓				
PASS - IADL						✓	✓	✓				✓			
DRUGS					✓	✓	✓	✓	✓	✓	✓				
S - TOFHLA						✓		✓	✓						
TOFHLA - R						✓					✓				
CHAS						✓	✓	✓		✓	✓				
FCCHL						✓	✓	✓		✓	✓				
LTMBSES															
SEAMS															
MMSE						✓	✓	✓	✓	✓	✓				
WCST						✓									
DSB						✓									
CVLT						✓									
Mini - Cog						✓	✓	✓							
Medi - Cog						✓	✓	✓							
MTS						✓	✓	✓	✓						
MOCA						✓	✓	✓	✓	✓	✓				
SBT						✓	✓	✓	✓						
TMT						✓									
MeDS						✓									
FOME						✓			✓						
NEI VFQ–25												✓		✓	
DLTV												✓	✓	✓	✓
PR test															✓
Randot Circles															
ETDRS												✓	✓		
Whisper test															
NVS															
REALM															
MASES															
MPED															
MSSS															
MSPSS															
PSS - Fr& PSS - Fa															

Tools	Sensory					Motivational						Environmental		
	Dark adaptation	Glare	Audition	Auditory acuity	Touch sensation	Trust in own ability	Efficiency in seeing benefits	Techno literacy	Health literacy	Shift in responsibilities from provider to patient not preferred	Integration of medication taking during daily activities	Social factors	Cost of medication adherence technologies	Home environment
MMS						✓	✓		✓					
RAT						✓	✓							
CSMS									✓					
MMAA												✓		
SMAT			✓									✓		
HOME - Rx revised						✓								✓
MedMaIDE									✓			✓		
Show Back									✓					
MedTake test									✓					
HOME - Rx						✓								
HMS									✓					
PASS - IADL			✓						✓					
DRUGS														
S - TOFHLA									✓					
TOFHLA - R									✓					
CHAS								✓	✓					
FCCHL							✓		✓					
LTMBSES						✓					✓	✓		
SEAMS						✓			✓	✓		✓		
MMSE														
WCST														
DSB														
CVLT														
Mini - Cog														
Medi - Cog														
MTS														
MOCA														
SBT														
TMT														
MeDS														
FOME														
NEI VFQ–25														
DLTV	✓	✓												
PR test														
Randot Circles														
ETDRS														
Whisper test			✓	✓										
NVS									✓					
REALM									✓					
MASES						✓								
MPED											✓			
MSSS												✓		
MSPSS												✓		
PSS - Fr& PSS - Fa												✓		

### Psychometric properties of assessment tools

It is important to establish psychometric properties of tools as they highlight each tool’s validity and reliability in clinical and research settings. If a tool lacks sufficient validity , the outcomes derived from the use of the tool cannot be confidently relied upon. Our review highlights a mixed picture regarding the psychometric properties of these tools. Instruments, such as the Self-medication Assessment Tool (SMAT) and Medication Management Ability Assessment (MMAA), demonstrate good psychometric properties through the assessment of their content and construct validity and with high scores in various reliability measures such as inter-rater reliability, test-retest reliability, and internal consistency [34, 49, 56]. However, other tools like the Cognitive Screen for Medication Self-Management (CSMS) showed potential issues with reliability, indicated by its low internal consistency scores [30]. Similarly, MedTake test only has only validity measures with a lack of various reliability measurements [53]. This variability indicates that while many tools have undergone some level of psychometric evaluation, there remains a gap in the comprehensive validation of these instruments. Future research should focus on addressing these gaps, particularly by expanding validation studies to include larger and more diverse populations, examining test-retest reliability, inter-rater reliability, and internal consistency more consistently, and exploring the practical implications of these tools in everyday clinical use.

### Clinical utility and implementation challenges

While identifying tools that are comprehensive is important, implementing such tools in clinical settings presents its own set of challenges. Most of the promising tools we identified are performance-based assessments, which healthcare professionals are responsible for administering. However, implementing these assessments in busy clinical environments can be challenging. Given that the administration times for these tools vary widely, from 5 minutes to 60 minutes, integrating them effectively into busy clinical workflows can be a hindrance to implementation. This is especially true when considering the average physician visit lasts approximately 15.7 minutes [100]. Consequently, use of comprehensive tools may be impractical within a clinical setting. However, clinicians can make use of these findings to selectively determine which tools are most suitable for the specific needs of the patients under their care.

### Limitations and real-world applicability of assessments

While the measurement of MMC provides valuable insights into an individual's ability to handle medications effectively, it's essential to recognize its limitations [20–23, 30, 34, 49, 56]. This assessment doesn't offer a comprehensive prediction of real-world medication-taking behavior [101, 102]. Medication non-adherence can be intentional or unintentional [6–9]. Intentional medication non-adherence, where individuals may consciously choose to deviate from prescribed regimens due to personal beliefs, concerns, or experiences with side effects, is not examined by these measurements [103]. However, incorporating MMC assessments into routine clinical practice allows clinicians to identify those who are unintentionally non-adherent and may benefit from person-specific assistance in managing their medications [21–24]. Such tailored interventions include patient education, simplified medication regimens, cognitive-behavioural therapy, and technology-based solutions to help manage medications [7]. Addressing barriers to MMC in older adults has the potential for long-term health benefits by improving overall well-being, reducing hospitalizations and complications associated with chronic conditions, while concurrently addressing the burden associated with managing medications [1, 3–5, 11, 21, 24].

## Strengths and limitations

### Strengths

One of the main strengths of this scoping review is the involvement of patient partners in the full text review and data extraction stages. Their valuable input not only provided insights into the needs and concerns of older adults regarding medication self-management, but also contributed to the identification of tools that were considered crucial for measuring diverse medication management components, drawing upon their personal lived experience with managing medications. Furthermore, by comprehensively identifying and comparing various tools that measure barriers to MMC, this scoping review contributes to the advancement of knowledge in the field of medication management in older adults. It serves as a reference point healthcare professionals can use for selecting tools to assess their patient's MMC. Researchers can use this information to select appropriate tools for their studies and to develop new tools that address specific barriers to MMC.

### Limitations

A limitation of this study is that it was limited to English language studies published between 2002 and 2022. There may be important studies that were excluded from this study due to language and time restrictions. Future research should consider including studies published in other languages to increase the comprehensiveness of the review. Additionally, although we searched six different databases using well-constructed search strategies, it is still possible that relevant studies were missed.

## Conclusion

This scoping review identified several validated tools to measure various challenges that older adults encounter with medication management. However, no one tool measures all five barriers (physical, cognitive, sensory, motivational, and environmental) to medication-taking at home. Therefore, a combination of tools is recommended to comprehensively measure these different aspects. The study's findings can aid healthcare professionals and researchers in selecting appropriate tools for assessing medication management capacity in older adults and enhancing the quality of care for this population. Nonetheless, despite the valuable insights from this review, the development of a comprehensive tool that addresses all these barriers is still necessary. Further research and development in this area is needed to provide healthcare professionals with a more efficient and holistic approach to assess medication management capacity.

### Supplementary Information

**Supplementary Material 1.**

## Data Availability

The datasets used and/or analysed during the current study are available from the corresponding author on reasonable request.

## Abbreviations

CSMS: Cognitive Screen for Medication Self-Management
CVLT: California Verbal Learning Test
CHAS: Comprehensive Health Activities Scale
DRUGS: Drug Regimen Unassisted Grading Scale
DLTV: Daily Living Tasks associated with Vision
DSB: Digit Span Backward
ETDRS: Early Treatment Diabetic Retinopathy Study eye chart
FCCHL: Functional, Communicative and Critical Health Literacy scale
FOME: Fuld Object-Memory Evaluation
HMS: Hopkins Medication Schedule
LTMBSES: Long-Term Medication Behavior Self-Efficacy Scale
MMAA: Medication Management Ability Assessment
MMC: Medication Management Capacity
MCI: Mild Cognitive Impairment
MMSE: Mini-Mental State Examination
MTS: Medication-Transfer screen
MedMaIDE: Medication Management Instrument for Deficiencies in the Elderly
MoCA: Montreal Cognitive Assessment
MSSS: Medication-Specific Social Support Questionnaire
MeDS: Measure of Drug Self-Management
MSPSS: Multidimensional Scale of Perceived Social Support
MPED: Martin and Park Environmental Demands Questionnaire
MMS: ManageMed Screening
MASES: Medication Administration Self-Efficacy Scale
NVS: The Newest Vital Sign
NEI VFQ-25: National Eye Institute Visual Function Questionnaire-25
PASS: Performance Assessment of Self-care Skills
PSS-Fr & PSS-Fa: Perceived Social Support from Friends and the Perceived Social Support from Family
PR test: Pelli*–*Robson test
REALM: Rapid Estimate of Adult Literacy in Medicine
RAT: Self-medication Risk Assessment Tool
S-TOFHLA: Short Test of Functional Health Literacy in Adults
SEAMS: Self-Efficacy for Appropriate Medication Use Scale
SMAT: Self-Medication Assessment Tool
SBT: Short Blessed Test
TMT: Trail-Making Test
TOFHLA-R: Test of Functional Health Literacy in Adults
WCST: Wisconsin Card Sorting Test
WHO: World Health Organization
