# Supplementary material for: Tools to measure barriers to medication management capacity in older adults: a scoping review
Source: BMC Geriatr. 2024 Mar 27;24:285. doi: 10.1186/s12877-024-04893-7 (PMC10967066; doi:10.1186/s12877-024-04893-7)
Supplement: Supplementary file 1 — Supplementary Material 1. [file 12877_2024_4893_MOESM1_ESM.docx]

**Additional file 1: Full Database Search Strategies**

**PubMed(MEDLINE):**

(medication therapy management[mesh] OR patient compliance[mesh] OR prescription drugs[mesh] OR prescription*[tiab] OR medication*[tiab] OR drug*[tiab] OR medicine*[tiab]) AND (self administration[mesh] OR self management[mesh] OR self efficacy[mesh] OR self care[mesh] OR "self administrat*"[tiab] OR "self manag*"[tiab] OR "self efficacy"[tiab] OR "self care"[tiab] OR “self treatment”[tiab] OR "self medicat*"[tiab]) AND (tool*[tiab] OR instrument*[tiab] OR framework*[tiab] OR assess*[tiab] OR surveys and questionnaires[mesh] OR survey*[tiab] OR questionnaire*[tiab] OR scale*[tiab] OR screen*[tiab] OR measur*[tiab] OR psychometrics[mesh] OR psychometric*[tiab]) AND (mental competency[mesh] OR competenc*[tiab] OR capacity[tiab] OR skill*[tiab] OR aptitude[mesh] OR aptitude[tiab] OR abilit*[tiab] OR hearing loss[mesh] OR auditory perception[mesh] OR “hearing loss”[tiab] OR “hearing impairment”[tiab] OR “impaired hearing”[tiab] OR “loss of hearing”[tiab] OR “hearing difficult*”[tiab] OR hypoacusis[tiab] OR hypacusia[tiab] OR hypacusis[tiab] OR hypoacousia[tiab] OR “transitory deafness*”[tiab] OR “transitory hearing loss”[tiab] OR “auditory acuity”[tiab] OR “hearing sensitivity”[tiab] OR “auditory perception”[tiab] OR deaf*[tiab] OR vision disorders[mesh] OR “vision disorder*”[tiab] OR “visual disorder*”[tiab] OR visual acuity[mesh] OR “visual acuity”[tiab] OR “vision impairment*”[tiab] OR “vision disturbance”[tiab] OR “visual disturbance”[tiab] OR “visual impairment*”[tiab] OR “visually impaired”[tiab] OR blind*[tiab] OR hemianopsia*[tiab] OR hemianopia[tiab] OR “vision defect*”[tiab] OR “colour blind*”[tiab] OR colourblind*[tiab] OR “color blind*”[tiab] OR colorblind*[tiab] OR diplopia[tiab] OR “double vision”[tiab] OR “seeing double”[tiab] OR photophobia[tiab] OR scotoma[tiab] OR “low vision”[tiab] OR “vision loss”[tiab] OR “poor vision”[tiab] OR “subnormal vision”[tiab] OR amblyopia[tiab] OR “lazy eye”[tiab] OR cataract[mesh] OR cataract*[tiab] OR “contrast detection”[tiab] OR “contrast sensitivity”[tiab] OR “sensory impairment”[tiab] OR “sensory loss”[tiab] OR “sensory dysfunction”[tiab] OR motor skills[mesh] OR “motor skill*”[tiab] OR “motor performance”[tiab] OR “psychomotor performance”[tiab] OR “motor function”[tiab] OR dexterity[tiab] OR agility[tiab] OR hand strength[mesh] OR strength[tiab] OR “hand eye coordination”[tiab] OR “eye hand coordination”[tiab] OR “eye hand control”[tiab] OR grip*[tiab] OR grasp*[tiab] OR “movement limitation*”[tiab] OR physical functional performance[mesh:noexp] OR "functional performance"[tiab] OR "functional impairment*"[tiab] OR "impaired functioning"[tiab] OR “functional disabilit*”[tiab] OR “functional decline”[tiab] OR “functional status”[tiab] OR “functional abilit*”[tiab] OR “physical abilit*”[tiab] OR “functional limitation*”[tiab] OR “functional restriction*”[tiab] OR “functional capacit*”[tiab] OR “physical function”[tiab] OR “functional independence”[tiab] OR “functional disease”[tiab] OR decision making[mesh] OR “decision making”[tiab] OR attention[mesh] OR attention[tiab] OR concentrat*[tiab] OR motivation[mesh:noexp] OR motivat*[tiab] OR thinking[mesh] OR think*[tiab] OR judgment[tiab] OR efficiency[mesh:noexp] OR efficien*[tiab] OR memory[mesh] OR memor*[tiab] OR cognition[mesh:noexp] OR cognition[tiab] OR cognitive dysfunction[mesh] OR “cognitive dysfunction”[tiab] OR “cognitive function”[tiab] OR “cognitive impairment”[tiab]) AND (aged[mesh] OR aged[tiab] OR elder*[tiab] OR geriatric*[tiab] OR "older people*"[tiab] OR "older person*"[tiab] OR "older adult*"[tiab] OR “older patient*”[tiab] OR senior*[tiab]) AND English[lang] AND 2002:2022[edat]

**Ovid Embase:**

1 exp medication therapy management/ 13513

2 exp medication compliance/ 40583

3 exp prescription drug/ 12132

4 (medication* or drug* or medicine* or prescription*).ti,ab. 3737293

5 or/1-4 3755108

6 exp drug self administration/ 12540

7 exp self care/ 93966

8 exp self concept/ 218328

9 exp self medication/ 11267

10 ("self administrat*" or "self manag*" or "self efficacy" or "self care" or "self treatment" or "self medicat*").ti,ab. 123841

11 or/6-10 354974

12 exp functional assessment/ 68464

13 exp geriatric assessment/ 19473

14 exp questionnaire/ 837162

15 exp psychometry/ 103495

16 (tool* or instrument* or framework* or assess* or survey* or questionnaire* or scale* or screen* or measur* or psychometric*).ti,ab. 11477933

17 or/12-16 11611896

18 exp mental capacity/ 89132

19 exp skill/ 99353

20 exp aptitude/ 4943

21 exp hearing impairment/ 108867

22 exp hearing acuity/ 3770

23 exp visual disorder/ 262981

24 exp visual acuity/ 142260

25 exp visual impairment/ 108437

26 exp blindness/ 47002

27 exp hemianopia/ 5182

28 exp color blindness/ 1806

29 exp diplopia/ 26242

30 exp photophobia/ 12210

31 exp scotoma/ 19538

32 exp low vision/ 4093

33 exp amblyopia/ 10810

34 exp cataract/ 64068

35 exp contrast sensitivity/ 11946

36 exp sensory dysfunction/ 643699

37 exp motor performance/ 84251

38 exp agility/ 2204

39 exp hand strength/ 34600

40 exp eye hand coordination/ 2045

41 exp grip strength/ 28648

42 exp physical performance/ 109239

43 exp functional disease/ 25652

44 *physical capacity/ 1946

45 exp decision making/ 427244

46 exp attention/ 299076

47 *motivation/ 30773

48 exp thinking/ 652873

49 exp memory/ 333611

50 *cognition/ 81989

51 exp cognitive defect/ 559028

52 (competenc* or capacity or skill* or aptitude or abilit* or "hearing loss" or "hearing impairment" or "impaired hearing" or "loss of hearing" or "hearing difficult*" or hypoacusis or hypacusia or hypacusis or hypoacousia or "transitory deafness" or "transitory hearing loss" or "auditory acuity" or "hearing sensitivity" or "auditory perception" or deaf* or "vision disorder*" or "visual disorder*" or "visual acuity" or "vision impairment" or "vision disturbance" or "visual disturbance" or "visual impairment" or "visually impaired" or blind* or hemianopsia or hemianopia or "vision defect*" or "colour blind*" or colourblind* or "color blind*" or colorblind* or diplopia or "double vision" or "seeing double" or photophobia or scotoma or "low vision" or "vision loss" or "poor vision" or "subnormal vision" or amblyopia or "lazy eye" or cataract* or "contrast detection" or "contrast sensitivity" or "sensory impairment" or "sensory loss" or "sensory dysfunction" or "motor skill*" or "motor performance" or "psychomotor performance" or "motor function" or dexterity or agility or strength or "hand eye coordination" or "eye hand coordination" or "eye hand control" or grip* or grasp* or "movement limitation*" or "functional performance" or "functional impairment*" or "impaired functioning" or "functional disabilit*" or "functional decline" or "functional status" or "functional abilit*" or "physical abilit*" or "functional limitation*" or "functional restriction*" or "functional capacit*" or "physical function" or "functional independence" or "functional disease" or "decision making" or attention or concentrat* or motivat* or think* or judgment or efficien* or memor* or cognition or "cognitive dysfunction" or "cognitive function" or "cognitive impairment").ti,ab. 8066842

53 or/18-52 9385101

54 exp aged/ 3382815

55 (aged or elder* or geriatric* or "older people" or "older person*" or "older adult*" or "older patient*" or senior*).ti,ab. 1471321

56 or/54-55 4213862

57 5 and 11 and 17 and 53 and 56 3537

58 limit 57 to (english language and yr="2002 -Current") 3164

**Ovid International Pharmaceutical Abstracts**

1 (medication* or drug* or medicine* or prescription*).ti,ab. 271958

2 ("self administrat*" or "self manag*" or "self efficacy" or "self care" or "self treatment" or "self medicat*").ti,ab. 2694

3 (tool* or instrument* or framework* or assess* or survey* or questionnaire* or scale* or screen* or measur* or psychometric*).ti,ab. 177686

4 (competenc* or capacity or skill* or aptitude or abilit* or "hearing loss" or "hearing impairment" or "impaired hearing" or "loss of hearing" or "hearing difficult*" or hypoacusis or hypacusia or hypacusis or hypoacousia or "transitory deafness" or "transitory hearing loss" or "auditory acuity" or "hearing sensitivity" or "auditory perception" or deaf* or "vision disorder*" or "visual disorder*" or "visual acuity" or "vision impairment" or "vision disturbance" or "visual disturbance" or "visual impairment" or "visually impaired" or blind* or hemianopsia or hemianopia or "vision defect*" or "colour blind*" or colourblind* or "color blind*" or colorblind* or diplopia or "double vision" or "seeing double" or photophobia or scotoma or "low vision" or "vision loss" or "poor vision" or "subnormal vision" or amblyopia or "lazy eye" or cataract* or "contrast detection" or "contrast sensitivity" or "sensory impairment" or "sensory loss" or "sensory dysfunction" or "motor skill*" or "motor performance" or "psychomotor performance" or "motor function" or dexterity or agility or strength or "hand eye coordination" or "eye hand coordination" or "eye hand control" or grip* or grasp* or "movement limitation*" or "functional performance" or "functional impairment*" or "impaired functioning" or "functional disabilit*" or "functional decline" or "functional status" or "functional abilit*" or "physical abilit*" or "functional limitation*" or "functional restriction*" or "functional capacit*" or "physical function" or "functional independence" or "functional disease" or "decision making" or attention or concentrat* or motivat* or think* or judgment or efficien* or memor* or cognition or "cognitive dysfunction" or "cognitive function" or "cognitive impairment").ti,ab. 167526

5 (aged or elder* or geriatric* or "older people" or "older person*" or "older adult*" or "older patient*" or senior*).ti,ab. 32061

6 1 and 2 and 3 and 4 and 5 46

7 limit 6 to (english language and yr="2002 -Current") 28

**EBSCOhost CINAHL**

| **#** | **Query** | **Limiters/Expanders** |
| --- | --- | --- |
| S55 | S5 AND S12 AND S22 AND S49 AND S52 | Limiters - Published Date: 20020101-20221231   Expanders - Apply equivalent subjects   Narrow by Language: - english   Search modes - Boolean/Phrase |
| S54 | S5 AND S12 AND S22 AND S49 AND S52 | Limiters - Published Date: 20020101-20221231 |
| S53 | S5 AND S12 AND S22 AND S49 AND S52 | Expanders - Apply equivalent subjects   Search modes - Boolean/Phrase |
| S52 | S50 OR S51 | Expanders - Apply equivalent subjects   Search modes - Boolean/Phrase |
| S51 | TI ( (aged OR elder* OR geriatric* OR "older people" OR "older person*" OR "older adult*" OR "older patient*" OR senior*) ) OR AB ( (aged OR elder* OR geriatric* OR "older people" OR "older person*" OR "older adult*" OR "older patient*" OR senior*) ) | Expanders - Apply equivalent subjects   Search modes - Boolean/Phrase |
| S50 | (MH "Aged+") | Expanders - Apply equivalent subjects   Search modes - Boolean/Phrase |
| S49 | S23 OR S24 OR S25 OR S26 OR S27 OR S28 OR S29 OR S30 OR S31 OR S32 OR S33 OR S34 OR S35 OR S36 OR S37 OR S38 OR S39 OR S40 OR S41 OR S42 OR S43 OR S44 OR S45 OR S46 OR S47 OR S48 | Expanders - Apply equivalent subjects   Search modes - Boolean/Phrase |
| S48 | TI ( (competenc* or capacity or skill* or aptitude or abilit* or "hearing loss" or "hearing impairment" or "impaired hearing" or "loss of hearing" or "hearing difficult*" or hypoacusis or hypacusia or hypacusis or hypoacousia or "transitory deafness" or "transitory hearing loss" or "auditory acuity" or "hearing sensitivity" or "auditory perception" or deaf* or "vision disorder*" or "visual disorder*" or "visual acuity" or "vision impairment" or "vision disturbance" or "visual disturbance" or "visual impairment" or "visually impaired" or blind* or hemianopsia or hemianopia or "vision defect*" or "colour blind*" or colourblind* or "color blind*" or colorblind* or diplopia or "double vision" or "seeing double" or photophobia or scotoma or "low vision" or "vision loss" or "poor vision" or "subnormal vision" or amblyopia or "lazy eye" or cataract* or "contrast detection" or "contrast sensitivity" or "sensory impairment" or "sensory loss" or "sensory dysfunction" or "motor skill*" or "motor performance" or "psychomotor performance" or "motor function" or dexterity or agility or strength or "hand eye coordination" or "eye hand coordination" or "eye hand control" or grip* or grasp* or "movement limitation*" or "functional performance" or "functional impairment*" or "impaired functioning" or "functional disabilit*" or "functional decline" or "functional status" or "functional abilit*" or "physical abilit*" or "functional limitation*" or "functional restriction*" or "functional capacit*" or "physical function" or "functional independence" or "functional disease" or "decision making" or attention or concentrat* or motivat* or think* or judgment or efficien* or memor* or cognition or "cognitive dysfunction" or "cognitive function" or "cognitive impairment") ) OR AB ( (competenc* or capacity or skill* or aptitude or abilit* or "hearing loss" or "hearing impairment" or "impaired hearing" or "loss of hearing" or "hearing difficult*" or hypoacusis or hypacusia or hypacusis or hypoacousia or "transitory deafness" or "transitory hearing loss" or "auditory acuity" or "hearing sensitivity" or "auditory perception" or deaf* or "vision disorder*" or "visual disorder*" or "visual acuity" or "vision impairment" or "vision disturbance" or "visual disturbance" or "visual impairment" or "visually impaired" or blind* or hemianopsia or hemianopia or "vision defect*" or "colour blind*" or colourblind* or "color blind*" or colorblind* or diplopia or "double vision" or "seeing double" or photophobia or scotoma or "low vision" or "vision loss" or "poor vision" or "subnormal vision" or amblyopia or "lazy eye" or cataract* or "contrast detection" or "contrast sensitivity" or "sensory impairment" or "sensory loss" or "sensory dysfunction" or "motor skill*" or "motor performance" or "psychomotor performance" or "motor function" or dexterity or agility or strength or "hand eye coordination" or "eye hand coordination" or "eye hand control" or grip* or grasp* or "movement limitation*" or "functional performance" or "functional impairment*" or "impaired functioning" or "functional disabilit*" or "functional decline" or "functional status" or "functional abilit*" or "physical abilit*" or "functional limitation*" or "functional restriction*" or "functional capacit*" or "physical function" or "functional independence" or "functional disease" or "decision making" or attention or concentrat* or motivat* or think* or judgment or efficien* or memor* or cognition or "cognitive dysfunction" or "cognitive function" or "cognitive impairment") ) | Expanders - Apply equivalent subjects   Search modes - Boolean/Phrase |
| S47 | (MH "Cognition") | Expanders - Apply equivalent subjects   Search modes - Boolean/Phrase |
| S46 | (MH "Memory+") | Expanders - Apply equivalent subjects   Search modes - Boolean/Phrase |
| S45 | (MH "Judgment") | Expanders - Apply equivalent subjects   Search modes - Boolean/Phrase |
| S44 | (MH "Thinking+") | Expanders - Apply equivalent subjects   Search modes - Boolean/Phrase |
| S43 | (MH "Motivation") | Expanders - Apply equivalent subjects   Search modes - Boolean/Phrase |
| S42 | (MH "Attention+") | Expanders - Apply equivalent subjects   Search modes - Boolean/Phrase |
| S41 | (MH "Decision Making+") | Expanders - Apply equivalent subjects   Search modes - Boolean/Phrase |
| S40 | (MH "Functional Status") | Expanders - Apply equivalent subjects   Search modes - Boolean/Phrase |
| S39 | (MH "Grip Strength") | Expanders - Apply equivalent subjects   Search modes - Boolean/Phrase |
| S38 | (MH "Agility") | Expanders - Apply equivalent subjects   Search modes - Boolean/Phrase |
| S37 | (MH "Psychomotor Performance+") | Expanders - Apply equivalent subjects   Search modes - Boolean/Phrase |
| S36 | (MH "Motor Skills+") | Expanders - Apply equivalent subjects   Search modes - Boolean/Phrase |
| S35 | (MH "Cataract") | Expanders - Apply equivalent subjects   Search modes - Boolean/Phrase |
| S34 | (MH "Amblyopia") | Expanders - Apply equivalent subjects   Search modes - Boolean/Phrase |
| S33 | (MH "Vision, Subnormal") | Expanders - Apply equivalent subjects   Search modes - Boolean/Phrase |
| S32 | (MH "Photophobia") | Expanders - Apply equivalent subjects   Search modes - Boolean/Phrase |
| S31 | (MH "Diplopia") | Expanders - Apply equivalent subjects   Search modes - Boolean/Phrase |
| S30 | (MH "Color Vision Defects") | Expanders - Apply equivalent subjects   Search modes - Boolean/Phrase |
| S29 | (MH "Blindness+") | Expanders - Apply equivalent subjects   Search modes - Boolean/Phrase |
| S28 | (MH "Visual Acuity") | Expanders - Apply equivalent subjects   Search modes - Boolean/Phrase |
| S27 | (MH "Vision Disorders+") | Expanders - Apply equivalent subjects   Search modes - Boolean/Phrase |
| S26 | (MH "Deafness+") | Expanders - Apply equivalent subjects   Search modes - Boolean/Phrase |
| S25 | (MH "Auditory Perception+") | Expanders - Apply equivalent subjects   Search modes - Boolean/Phrase |
| S24 | (MH "Hearing Disorders+") | Expanders - Apply equivalent subjects   Search modes - Boolean/Phrase |
| S23 | (MH "Aptitude") | Expanders - Apply equivalent subjects   Search modes - Boolean/Phrase |
| S22 | S13 OR S14 OR S15 OR S16 OR S17 OR S18 OR S19 OR S20 OR S21 | Expanders - Apply equivalent subjects   Search modes - Boolean/Phrase |
| S21 | TI ( (tool* OR instrument* OR framework* OR assess* OR survey* OR questionnaire* OR scale* OR screen* OR measur* OR psychometric*) ) OR AB ( (tool* OR instrument* OR framework* OR assess* OR survey* OR questionnaire* OR scale* OR screen* OR measur* OR psychometric*) ) | Expanders - Apply equivalent subjects   Search modes - Boolean/Phrase |
| S20 | (MH "Psychometrics") | Expanders - Apply equivalent subjects   Search modes - Boolean/Phrase |
| S19 | (MH "Scales") | Expanders - Apply equivalent subjects   Search modes - Boolean/Phrase |
| S18 | (MH "Questionnaires+") | Expanders - Apply equivalent subjects   Search modes - Boolean/Phrase |
| S17 | (MH "Surveys+") | Expanders - Apply equivalent subjects   Search modes - Boolean/Phrase |
| S16 | (MH "Functional Assessment+") | Expanders - Apply equivalent subjects   Search modes - Boolean/Phrase |
| S15 | (MH "Geriatric Functional Assessment") | Expanders - Apply equivalent subjects   Search modes - Boolean/Phrase |
| S14 | (MH "Research Instruments+") | Expanders - Apply equivalent subjects   Search modes - Boolean/Phrase |
| S13 | (MH "Clinical Assessment Tools+") | Expanders - Apply equivalent subjects   Search modes - Boolean/Phrase |
| S12 | S6 OR S7 OR S8 OR S9 OR S10 OR S11 | Expanders - Apply equivalent subjects   Search modes - Boolean/Phrase |
| S11 | TI ( ("self administrat*" OR "self manag*" OR "self efficacy" OR "self care" OR “self treatment” OR "self medicat*") ) OR AB ( ("self administrat*" OR "self manag*" OR "self efficacy" OR "self care" OR “self treatment” OR "self medicat*") ) | Expanders - Apply equivalent subjects   Search modes - Boolean/Phrase |
| S10 | (MH "Self Medication") | Expanders - Apply equivalent subjects   Search modes - Boolean/Phrase |
| S9 | (MH "Self Care+") | Expanders - Apply equivalent subjects   Search modes - Boolean/Phrase |
| S8 | (MH "Self-Efficacy") | Expanders - Apply equivalent subjects   Search modes - Boolean/Phrase |
| S7 | (MH "Self-Management") | Expanders - Apply equivalent subjects   Search modes - Boolean/Phrase |
| S6 | (MH "Self Administration+") | Expanders - Apply equivalent subjects   Search modes - Boolean/Phrase |
| S5 | S1 OR S2 OR S3 OR S4 | Expanders - Apply equivalent subjects   Search modes - Boolean/Phrase |
| S4 | TI ( (prescription* OR medication* OR drug* OR medicine*) ) OR AB ( (prescription* OR medication* OR drug* OR medicine*) ) | Expanders - Apply equivalent subjects   Search modes - Boolean/Phrase |
| S3 | (MH "Drugs, Prescription+") | Expanders - Apply equivalent subjects   Search modes - Boolean/Phrase |
| S2 | (MH "Medication Compliance") | Expanders - Apply equivalent subjects   Search modes - Boolean/Phrase |
| S1 | (MH “Medication Management”) | Expanders - Apply equivalent subjects   Search modes - Boolean/Phrase |

**APA PsycINFO**

((((((title: (medication*))) OR ((title: (drug*))) OR ((title: (medicine*))) OR ((title: (prescription*))))) OR ((((abstract: (medication*))) OR ((abstract: (drug*))) OR ((abstract: (medicine*))) OR ((abstract: (prescription*)))))) AND (((((title: ("self administrat*"))) OR ((title: ("self manag*"))) OR ((title: ("self efficacy"))) OR ((title: ("self care"))) OR ((title: ("self treatment"))) OR ((title: ("self medicat*"))))) OR ((((abstract: ("self administrat*"))) OR ((abstract: ("self manag*"))) OR ((abstract: ("self efficacy"))) OR ((abstract: ("self care"))) OR ((abstract: ("self treatment"))) OR ((abstract: ("self medicat*")))))) AND (((((title: (tool*))) OR ((title: (instrument*))) OR ((title: (framework*))) OR ((title: (assess*))) OR ((title: (survey*))) OR ((title: (questionnaire*))) OR ((title: (scale*))) OR ((title: (screen*))) OR ((title: (measur*))) OR ((title: (psychometric*))))) OR ((((abstract: (tool*))) OR ((abstract: (instrument*))) OR ((abstract: (framework*))) OR ((abstract: (assess*))) OR ((abstract: (survey*))) OR ((abstract: (questionnaire*))) OR ((abstract: (scale*))) OR ((abstract: (screen*))) OR ((abstract: (measur*))) OR ((abstract: (psychometric*)))))) AND (((((title: (competenc*))) OR ((title: (capacity))) OR ((title: (skill*))) OR ((title: (aptitude))) OR ((title: (abilit*))) OR ((title: ("hearing loss"))) OR ((title: ("hearing impairment"))) OR ((title: ("impaired hearing"))) OR ((title: ("loss of hearing"))) OR ((title: ("hearing difficult*"))) OR ((title: (hypoacusis))) OR ((title: (hypacusia))) OR ((title: (hypacusis))) OR ((title: (hypoacousia))) OR ((title: ("transitory deafness"))) OR ((title: ("transitory hearing loss"))) OR ((title: ("auditory acuity"))) OR ((title: ("hearing sensitivity"))) OR ((title: ("auditory perception"))) OR ((title: (deaf*))) OR ((title: ("vision disorder*"))) OR ((title: ("visual disorder*"))) OR ((title: ("visual acuity"))) OR ((title: ("vision impairment"))) OR ((title: ("vision disturbance"))) OR ((title: ("visual disturbance"))) OR ((title: ("visual impairment"))) OR ((title: ("visually impaired"))) OR ((title: (blind*))) OR ((title: (hemianopsia))) OR ((title: (hemianopia))) OR ((title: ("vision defect*"))) OR ((title: ("colour blind*"))) OR ((title: (colourblind*))) OR ((title: ("color blind*"))) OR ((title: (colorblind*))) OR ((title: (diplopia))) OR ((title: ("double vision"))) OR ((title: ("seeing double"))) OR ((title: (photophobia))) OR ((title: (scotoma))) OR ((title: ("low vision"))) OR ((title: ("vision loss"))) OR ((title: ("poor vision"))) OR ((title: ("subnormal vision"))) OR ((title: (amblyopia))) OR ((title: ("lazy eye"))) OR ((title: (cataract*))) OR ((title: ("contrast detection"))) OR ((title: ("contrast sensitivity"))) OR ((title: ("sensory impairment"))) OR ((title: ("sensory loss"))) OR ((title: ("sensory dysfunction"))) OR ((title: ("motor skill*"))) OR ((title: ("motor performance"))) OR ((title: ("psychomotor performance"))) OR ((title: ("motor function"))) OR ((title: (dexterity))) OR ((title: (agility))) OR ((title: (strength))) OR ((title: ("hand eye coordination"))) OR ((title: ("eye hand coordination"))) OR ((title: ("eye hand control"))) OR ((title: (grip*))) OR ((title: (grasp*))) OR ((title: ("movement limitation*"))) OR ((title: ("functional performance"))) OR ((title: ("functional impairment*"))) OR ((title: ("impaired functioning"))) OR ((title: ("functional disabilit*"))) OR ((title: ("functional decline"))) OR ((title: ("functional status"))) OR ((title: ("functional abilit*"))) OR ((title: ("physical abilit*"))) OR ((title: ("functional limitation*"))) OR ((title: ("functional restriction*"))) OR ((title: ("functional capacit*"))) OR ((title: ("physical function"))) OR ((title: ("functional independence"))) OR ((title: ("functional disease"))) OR ((title: ("decision making"))) OR ((title: (attention))) OR ((title: (concentrat*))) OR ((title: (motivat*))) OR ((title: (think*))) OR ((title: (judgment))) OR ((title: (efficien*))) OR ((title: (memor*))) OR ((title: (cognition))) OR ((title: ("cognitive dysfunction"))) OR ((title: ("cognitive function"))) OR ((title: ("cognitive impairment"))))) OR ((((abstract: (competenc*))) OR ((abstract: (capacity))) OR ((abstract: (skill*))) OR ((abstract: (aptitude))) OR ((abstract: (abilit*))) OR ((abstract: ("hearing loss"))) OR ((abstract: ("hearing impairment"))) OR ((abstract: ("impaired hearing"))) OR ((abstract: ("loss of hearing"))) OR ((abstract: ("hearing difficult*"))) OR ((abstract: (hypoacusis))) OR ((abstract: (hypacusia))) OR ((abstract: (hypacusis))) OR ((abstract: (hypoacousia))) OR ((abstract: ("transitory deafness"))) OR ((abstract: ("transitory hearing loss"))) OR ((abstract: ("auditory acuity"))) OR ((abstract: ("hearing sensitivity"))) OR ((abstract: ("auditory perception"))) OR ((abstract: (deaf*))) OR ((abstract: ("vision disorder*"))) OR ((abstract: ("visual disorder*"))) OR ((abstract: ("visual acuity"))) OR ((abstract: ("vision impairment"))) OR ((abstract: ("vision disturbance"))) OR ((abstract: ("visual disturbance"))) OR ((abstract: ("visual impairment"))) OR ((abstract: ("visually impaired"))) OR ((abstract: (blind*))) OR ((abstract: (hemianopsia))) OR ((abstract: (hemianopia))) OR ((abstract: ("vision defect*"))) OR ((abstract: ("colour blind*"))) OR ((abstract: (colourblind*))) OR ((abstract: ("color blind*"))) OR ((abstract: (colorblind*))) OR ((abstract: (diplopia))) OR ((abstract: ("double vision"))) OR ((abstract: ("seeing double"))) OR ((abstract: (photophobia))) OR ((abstract: (scotoma))) OR ((abstract: ("low vision"))) OR ((abstract: ("vision loss"))) OR ((abstract: ("poor vision"))) OR ((abstract: ("subnormal vision"))) OR ((abstract: (amblyopia))) OR ((abstract: ("lazy eye"))) OR ((abstract: (cataract*))) OR ((abstract: ("contrast detection"))) OR ((abstract: ("contrast sensitivity"))) OR ((abstract: ("sensory impairment"))) OR ((abstract: ("sensory loss"))) OR ((abstract: ("sensory dysfunction"))) OR ((abstract: ("motor skill*"))) OR ((abstract: ("motor performance"))) OR ((abstract: ("psychomotor performance"))) OR ((abstract: ("motor function"))) OR ((abstract: (dexterity))) OR ((abstract: (agility))) OR ((abstract: (strength))) OR ((abstract: ("hand eye coordination"))) OR ((abstract: ("eye hand coordination"))) OR ((abstract: ("eye hand control"))) OR ((abstract: (grip*))) OR ((abstract: (grasp*))) OR ((abstract: ("movement limitation*"))) OR ((abstract: ("functional performance"))) OR ((abstract: ("functional impairment*"))) OR ((abstract: ("impaired functioning"))) OR ((abstract: ("functional disabilit*"))) OR ((abstract: ("functional decline"))) OR ((abstract: ("functional status"))) OR ((abstract: ("functional abilit*"))) OR ((abstract: ("physical abilit*"))) OR ((abstract: ("functional limitation*"))) OR ((abstract: ("functional restriction*"))) OR ((abstract: ("functional capacit*"))) OR ((abstract: ("physical function"))) OR ((abstract: ("functional independence"))) OR ((abstract: ("functional disease"))) OR ((abstract: ("decision making"))) OR ((abstract: (attention))) OR ((abstract: (concentrat*))) OR ((abstract: (motivat*))) OR ((abstract: (think*))) OR ((abstract: (judgment))) OR ((abstract: (efficien*))) OR ((abstract: (memor*))) OR ((abstract: (cognition))) OR ((abstract: ("cognitive dysfunction"))) OR ((abstract: ("cognitive function"))) OR ((abstract: ("cognitive impairment")))))) AND (((((title: (aged))) OR ((title: (elder*))) OR ((title: (geriatric*))) OR ((title: ("older people"))) OR ((title: ("older person*"))) OR ((title: ("older adult*"))) OR ((title: ("older patient*"))) OR ((title: (senior*))))) OR ((((abstract: (aged))) OR ((abstract: (elder*))) OR ((abstract: (geriatric*))) OR ((abstract: ("older people"))) OR ((abstract: ("older person*"))) OR ((abstract: ("older adult*"))) OR ((abstract: ("older patient*"))) OR ((abstract: (senior*))))))) AND ((Language: (english))) AND Year: 2002 To 2022

**Scopus**

( TITLE-ABS ( ( medication*  OR  drug*  OR  medicine*  OR  prescription* ) )  AND  TITLE-ABS ( ( "self administrat*"  OR  "self manag*"  OR  "self efficacy"  OR  "self care"  OR  "self treatment"  OR  "self medicat*" ) )  AND  TITLE-ABS ( ( tool*  OR  instrument*  OR  framework*  OR  assess*  OR  survey*  OR  questionnaire*  OR  scale*  OR  screen*  OR  measur*  OR  psychometric* ) )  AND  TITLE-ABS ( ( competenc*  OR  capacity  OR  skill*  OR  aptitude  OR  abilit*  OR  "hearing loss"  OR  "hearing impairment"  OR  "impaired hearing"  OR  "loss of hearing"  OR  "hearing difficult*"  OR  hypoacusis  OR  hypacusia  OR  hypacusis  OR  hypoacousia  OR  "transitory deafness"  OR  "transitory hearing loss"  OR  "auditory acuity"  OR  "hearing sensitivity"  OR  "auditory perception"  OR  deaf*  OR  "vision disorder*"  OR  "visual disorder*"  OR  "visual acuity"  OR  "vision impairment"  OR  "vision disturbance"  OR  "visual disturbance"  OR  "visual impairment"  OR  "visually impaired"  OR  blind*  OR  hemianopsia  OR  hemianopia  OR  "vision defect*"  OR  "colour blind*"  OR  colourblind*  OR  "color blind*"  OR  colorblind*  OR  diplopia  OR  "double vision"  OR  "seeing double"  OR  photophobia  OR  scotoma  OR  "low vision"  OR  "vision loss"  OR  "poor vision"  OR  "subnormal vision"  OR  amblyopia  OR  "lazy eye"  OR  cataract*  OR  "contrast detection"  OR  "contrast sensitivity"  OR  "sensory impairment"  OR  "sensory loss"  OR  "sensory dysfunction"  OR  "motor skill*"  OR  "motor performance"  OR  "psychomotor performance"  OR  "motor function"  OR  dexterity  OR  agility  OR  strength  OR  "hand eye coordination"  OR  "eye hand coordination"  OR  "eye hand control"  OR  grip*  OR  grasp*  OR  "movement limitation*"  OR  "functional performance"  OR  "functional impairment*"  OR  "impaired functioning"  OR  "functional disabilit*"  OR  "functional decline"  OR  "functional status"  OR  "functional abilit*"  OR  "physical abilit*"  OR  "functional limitation*"  OR  "functional restriction*"  OR  "functional capacit*"  OR  "physical function"  OR  "functional independence"  OR  "functional disease"  OR  "decision making"  OR  attention  OR  concentrat*  OR  motivat*  OR  think*  OR  judgment  OR  efficien*  OR  memor*  OR  cognition  OR  "cognitive dysfunction"  OR  "cognitive function"  OR  "cognitive impairment" ) )  AND  TITLE-ABS ( ( aged  OR  elder*  OR  geriatric*  OR  "older people"  OR  "older person*"  OR  "older adult*"  OR  "older patient*"  OR  senior* ) )  AND  LANGUAGE ( english ) )  AND  PUBYEAR  >  2001
